# Diurnal Rhythms in the Red Seaweed *Gracilariopsis chorda* are Characterized by Unique Regulatory Networks of Carbon Metabolism

**DOI:** 10.1093/molbev/msae012

**Published:** 2024-01-24

**Authors:** JunMo Lee, Ji Hyun Yang, Andreas P M Weber, Debashish Bhattacharya, Woe-Yeon Kim, Hwan Su Yoon

**Affiliations:** Department of Oceanography, Kyungpook National University, Daegu 41566, Korea; Kyungpook Institute of Oceanography, Kyungpook National University, Daegu 41566, Korea; Department of Biological Sciences, Sungkyunkwan University, Suwon 16419, Korea; Institute of Plant Biochemistry, Cluster of Excellence on Plant Science (CEPLAS), Heinrich Heine University, 40225 Düsseldorf, Germany; Department of Biochemistry and Microbiology, Rutgers University, New Brunswick, NJ 08901, USA; Division of Applied Life Science (BK21 four), Research Institute of Life Science, Gyeongsang National University, Jinju 52828, Korea; Department of Biological Sciences, Sungkyunkwan University, Suwon 16419, Korea

**Keywords:** *Gracilariopsis chorda*, rhythmic gene, cryptochrome, cytosolic carbon metabolism, horizontal gene transfers

## Abstract

Cellular and physiological cycles are driven by endogenous pacemakers, the diurnal and circadian rhythms. Key functions such as cell cycle progression and cellular metabolism are under rhythmic regulation, thereby maintaining physiological homeostasis. The photoreceptors phytochrome and cryptochrome, in response to light cues, are central input pathways for physiological cycles in most photosynthetic organisms. However, among Archaeplastida, red algae are the only taxa that lack phytochromes. Current knowledge about oscillatory rhythms is primarily derived from model species such as *Arabidopsis thaliana* and *Chlamydomonas reinhardtii* in the Viridiplantae, whereas little is known about these processes in other clades of the Archaeplastida, such as the red algae (Rhodophyta). We used genome-wide expression profiling of the red seaweed *Gracilariopsis chorda* and identified 3,098 rhythmic genes. Here, we characterized possible cryptochrome-based regulation and photosynthetic/cytosolic carbon metabolism in this species. We found a large family of cryptochrome genes in *G*. *chorda* that display rhythmic expression over the diurnal cycle and may compensate for the lack of phytochromes in this species. The input pathway gates regulatory networks of carbon metabolism which results in a compact and efficient energy metabolism during daylight hours. The system in *G*. *chorda* is distinct from energy metabolism in most plants, which activates in the dark. The green lineage, in particular, land plants, balance water loss and CO_2_ capture in terrestrial environments. In contrast, red seaweeds maintain a reduced set of photoreceptors and a compact cytosolic carbon metabolism to thrive in the harsh abiotic conditions typical of intertidal zones.

## Introduction

Physiological and behavioral cycles fluctuate over 24 h, even in the absence of external stimuli. Diverse organisms including cyanobacteria (Piechura et al. 2017; Taton et al. 2020), animals (Whitmore et al. 1998; Jones et al. 2013; Ashbrook et al. 2020; Krylov et al. 2021), insects (Nitabach and Taghert 2008), and land plants (Harmer 2009) display circadian rhythms. Day and night transitions and photoperiodic oscillations determine a large proportion of physiological regulation thus it is particularly important in photosynthetic organisms (Brown et al. 2012; Dodd et al. 2013; de Dios and Gessler 2018). Physiological cycles and their regulatory mechanisms in algae and plants have largely been studied in model species such as *Arabidopsis thaliana* and the green algae *Chlamydomonas reinhardtii* and *Ostreococcus tauri* (Matsuo et al. 2008; Corellou et al. 2009; De Caluwé et al. 2016; Linde et al. 2017). In contrast, very little is known about diurnal rhythms and their impacts on physiology in red algae (Rhodophyta), which with green and glaucophyte algae, comprise the photosynthetic members of the Archaeplastida.

The photoreceptors, phytochrome (PHY) and cryptochrome (CRY) play central roles in the input pathways of circadian oscillation based on light cues (Hughes et al. 2012; Lopez et al. 2021; Liu et al. 2022), however, the *PHY* gene family is absent in red algae (Duanmu et al. 2014). Therefore, the oscillatory mechanisms in algae and plants differ and some shared circadian genes may have evolved through convergent evolution (Matsuo and Ishiura 2011; Linde et al. 2017; Serrano-Bueno et al. 2017). CRYs are blue light-sensitive proteins that are homologs of DNA photolyases (ultraviolet-damaged DNA repair enzymes; Fortunato et al. 2015). Despite this shared ancestry, CRYs have diverse functions in signaling and interaction, regulating physiological cycles such as circadian and diurnal rhythms (Fortunato et al. 2015; Lopez et al. 2021). CRYs are classified into three major groups: plant, animal, and DASH (*Drosophila*-*Arabidopsis*-*Synechocystis*-*Homo*) families (Kianianmomeni and Hallmann 2014).

Plant CRYs (pCRYs), such as those found in *Arabidopsis thaliana*, regulate the expression of nuclear-encoded genes involved in photoresponsive regulatory mechanisms in green lineages, including photosynthesis (e.g. Calvin cycle-related genes), growth, and development, and life cycle progression (Ohgishi et al. 2004; Fortunato et al. 2015; Wang and Lin 2020). In *Arabidopsis*, more than 30 pCRY-interacting proteins have been identified (Wang and Lin 2020), and light-activated pCRYs directly interact with the complex of Constitutive Photomorphogenic 1 (COP1) and Suppressor of PHY A (SPA). This interaction inhibits the E3 ubiquitin ligase activity of the COP1/SPA complex which degrades light-inducible transcription factors for photomorphogenesis in the dark (Fortunato et al. 2015; Wang and Lin 2020).

In plants, myeloblastosis-like transcription factors, *CIRCADIAN CLOCK-ASSOCIATED 1* (*CCA1*) and *LATE ELONGATED HYPOCOTYL* (*LHY*), which have peak gene expression at dawn and the most reduced at dusk, repress the transcription of clock-associated genes like *FLAVIN-BINDING, KELCH REPEAT AND F BOX 1* (*FKF1*), a key regulator in photoperiodic flowering (Schaffer et al. 1998, Wang and Tobin 1998; Imaizumi et al 2005; Sawa et al. 2007; Nakamichi 2020; Lopez et al. 2021). *REVEILLE8* (*RVE8*), homologous to *CCA1*/*LHY*, also shows a gene expression peak at dawn and acts as an activator of flowering along with the *NIGHT LIGHT-INDUCIBLE AND CLOCK-REGULATED* (*LNK*) genes (Farinas and Mas 2011; Nakamichi 2020). Homologs of the transcription factor *CCA1*/*LHY* are present in green algae, although little is known about the associated genes (e.g. *FKF1* and *LNK*s) in these species (Linde et al. 2017; Serrano-Bueno et al. 2017).

Animal CRYs (aCRYs) control cellular homeostasis in animals, regulating processes such as inflammation, the DNA damage response, and diverse features of metabolism (Michael et al. 2017). Multiple functions of DASH-CRYs have been reported from cyanobacteria (transcriptional repressor, photosystem II repair; Brudler et al. 2003; Vass et al. 2014), fungi (development, circadian clock; Froehlich et al. 2010; Castrillo et al. 2013), plants (organelle functions with dual targeting signals; Kleine et al. 2003), and algae (DNA repair, growth, potential photoreceptor, balances of photosynthetic machinery; Asimgil and Kavakli 2012; Yang et al. 2020; Rredhi et al. 2021). From the central input systems, pathways, including photosynthesis and CO_2_ fixation (e.g. the Calvin–Benson–Bassham cycle) are under rhythmic regulation vis-à-vis processes such as transcription-translation and post-translational feedback loops (Brown et al. 2012; Dodd et al. 2013; McClung 2019).

Central carbon metabolism (e.g. glycolysis/gluconeogenesis) is an intermediate step in both photosynthetic carbon assimilation and energy metabolism (Walker et al. 2021). Pyruvate-related metabolism is important not only for glycolysis/gluconeogenesis, but also for cytosolic carbon (e.g. carbon concentration, and interconversion of C_3_ to C_4_ organic acids) and mitochondrial metabolism. Phosphoenolpyruvate (PEP) carboxylase (PEPC; PEP + HCO_3_  ^−^ → OAA + Pi) plays crucial roles in carbon and nitrogen metabolism, including the intermediate step in the TCA cycle (Chollet et al. 1996; Shi et al. 2015). Carbon flow is closely linked to mitochondrial energy (i.e. adenosine triphosphate [ATP]) metabolism *via* oxidative phosphorylation, under both light and dark conditions (Raghavendra and Padmasree 2003). The cycling of mitochondrial energy production is directly related to the response to reactive oxygen species (ROS) (de Goede et al. 2018). ROS production in photosynthetic cells can be managed by “malate circulation” between plastids and mitochondria (Zhao et al. 2020), as well as through cell cycle controls (Miyagishima et al. 2014).

There are many different types of metabolite transporters involved in photosynthesis and energy metabolism in algae that have diversified *via* serial endosymbiosis (Facchinelli and Weber 2011). Some of these genes adapted to the limiting light levels and low CO_2_ gas diffusion rate in aquatic environments (Raven 1970; Zeebe 2011). These differences have resulted in divergent physiological responses in algae with respect to genes, photoreceptors, and their modulators (Noordally and Millar 2014). However, red algal systems are poorly studied even though a large proportion of photosynthetic eukaryotes (i.e. cryptophytes, haptophytes, stramenopiles, and apicomplexans) contain a red-alga-derived plastid (Bhattacharya et al. 2004). Here, we studied gene expression over the diurnal cycle in the red seaweed *Gracilariopsis chorda* (class Florideophyceae) and described CRY-derived regulation and photosynthetic/cytosolic carbon metabolism in this species. We find that *G*. *chorda* has a compact and efficient energy metabolism that is distinct from C_3_, C_4_, or Crassulacean acid metabolism (CAM) in plants. We postulate that rhythmic regulation of physiological cycles in eukaryotes could plausibly originate *via* multiple, independent horizontal gene transfers (HGTs).

## Results and Discussion

### Rhythmic Gene Expression in the Red Seaweed *Gracilariopsis chorda*

To study the rhythmic regulation of physiological cycles in *G*. *chorda*, algal samples were exposed to a day/night (DN) cycle for 24 h (12 h-light:12 h-dark) and thereafter, continuous light (CL) for 24 h. We collected algal samples each 6 h from DN (DN4, DN10, DN16, DN22) to CL (CL4, CL10, CL16, and CL22) conditions for RNA-seq analysis (data available at SRR21594546—SRR21594568; details in Methods). The DN samples can be used to study diurnal gene expression that underlies diverse physiological traits in *G*. *chorda*. The CL samples comprise two periods as follows: (i) CL4 and CL10, which provides additional insights into light-based cues for physiological cycles after the DN period (Hughes et al. 2012; Liu et al. 2022), and (ii) CL16 and CL22, which abolishes the DN transition and can provide insights into the expression of genes that are independent of the initial dark period. Based on the *G*. *chorda* gene expression patterns, we identified 3,098 rhythmic genes in this red seaweed (supplementary table S1, Supplementary Material online).

**Fig. 1.**
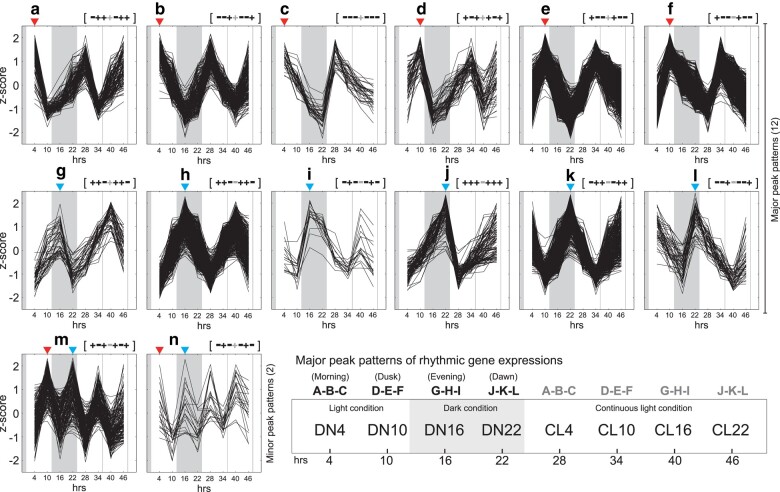
Rhythmic gene expression patterns in the red seaweed *Gracilariopsis chorda*. The dark areas in the plots are nighttime hours. The major peak patterns are labeled as pattern-a to pattern-l, grouped into morning-phased (a-b-c), dusk-phased (d-e-f), evening-phased (g-h-i), and dawn-phased (j-k-l) genes. The two fluctuating rhythmic gene expression patterns are considered minor peak patterns (m and n). The relative gene expression patterns are marked with the “+” and “–” symbols, indicating “increase” and “decrease” in gene expression when compared to the previous time point, respectively (see Materials and Methods).

Rhythmic gene expression patterns were divided into 12 major and two minor patterns that show diurnal fluctuation (Fig. 1). The up- and down-regulated genes from DN4 are shown with “+” and “−”, respectively, which indicate relative gene expression patterns compared to the previous time point (see Materials and Methods). Based on these results, we defined morning-phased (a-b-c; peak at DN4), dusk-phased (d-e-f; DN10), evening-phased (g-h-i; DN16), and dawn-phased (j-k-l; DN22) rhythmic genes (Fig. 1). However, the trough time was different within each group; the trough time in pattern-a is at DN10, which is earlier than the others (i.e. pattern-b at DN16, and pattern-c at DN22) within morning-phased group (Fig. 1). Therefore, patterns a, d, g, and j show gradual increase and then rapid decrease in gene expression. In contrast, patterns c, f, i, and l show rapid increase and gradual decrease. The patterns b, e, h, and k show a moderate cycle of increase and decrease in gene expression. We postulate that gene expression patterns are driven by sequential regulation (e.g. feedback loop) of rhythmic genes in functional groups. It is possible that the rhythmic genes we have identified in *G*. *chorda* may include circadian oscillators, but our current data do not allow us robustly to test this idea. In other words, to characterize true circadian gene expression in *G*. *chorda*, additional sampling from CL conditions for at least 24 h, which are not part of the previous diurnal cycle, is required. The minor patterns are indicated as fluctuating rhythmic gene expression and the peaks are present before (pattern-m) or after (pattern-n) both DN and night/day transitions (Fig. 1). These genes are potentially involved in preparation for the transitional points in the *G*. *chorda* cycle. Additional experiments are needed to address this hypothesis. Therefore, we focus here on diurnal rhythms in *G*. *chorda*.

### Cryptochrome Genes in *G*. *chorda*

**Fig. 2.**
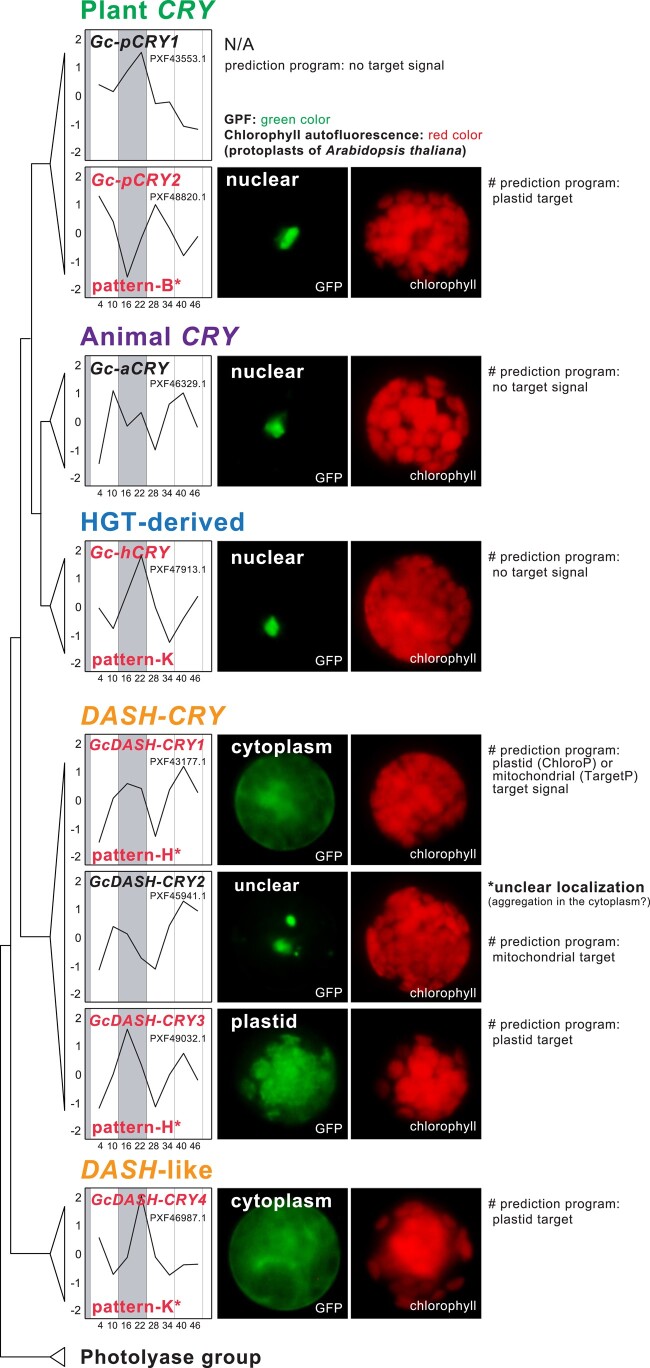
Gene expression of cryptochromes (*CRY*s) in *G*. *chorda*. The horizontal axis indicates each sampling point (hours), and the vertical axis indicates the *z*-score of the target gene expression. The red- and black-colored gene names indicate rhythmic and nonrhythmic genes, respectively (asterisk: significant correlation in rhythmic pattern). The in-frame GFP fusion of the *Gc-pCRY1* (PXF43553.1) gene was not available (N/A) in this study (see Materials and Methods). The tree, resulting from a ML phylogenetic analysis of *CRY*s, is shown in a simplified format (supplementary fig. S1, Supplementary Material online). The images on the right side are results of subcellular localization of CRYs in *G*. *chorda* when expressed in the heterologous system of *A*. *thaliana*. Bioinformatic predictions of subcellular localization using CRYs in *G*. *chorda* are provided (See Methods).

*G*. *chorda* contains multiple *CRY* genes; 2 plant-like (*pCRY*; *Gc-pCRY1* and *Gc-pCRY2*), an animal-like (*aCRY*; *Gc-aCRY*), 4 *DASH* or *DASH*-like (*DASH-CRY*; *GcDASH-CRY1*—*GcDASH-CRY4*) representatives, as well as an HGT-derived (*Gc-hCRY*) *CRY*-like gene (Fig. 2, supplementary fig. S1, Supplementary Material online). The *CRY* families in algae and plants act as regulators of the circadian clock and/or as enzymes to repair UV-induced DNA damage (Asimgil and Kavakli 2012; Kianianmomeni and Hallmann 2014; Fortunato et al. 2015; Zou et al. 2017; Kiontke et al. 2020; Petersen et al. 2021; Rredhi et al. 2021). pCRYs also function as regulators of organelle-encoded gene expression, e.g. in plastids, *via* regulation of nuclear-encoded prokaryotic transcription factors (Thum et al. 2001; Ichikawa et al. 2004; Ohgishi et al. 2004; Noordally et al. 2013; Fortunato et al. 2015). We postulate that the large family of *CRY*s in *G*. *chorda* compensates for the absence of phytochromes in this species and, potentially, in other red algae.

To study the role of these *CRY*s in *G*. *chorda* biology, we examined their expression under diurnal and CL conditions. We found that 5/8 *CRY* genes and key photosynthetic genes in *G*. *chorda* are under rhythmic regulation (Fig. 2, supplementary table S2, Supplementary Material online). In *G*. *chorda*, only *Gc-pCRY2* among the two *pCRY* genes exhibits rhythmic gene expression (Fig. 2) belonging to a morning-phased gene group (pattern-B in Fig. 1). This pattern is similar to the rhythmic expression profile of plastid-targeted light-harvesting proteins (e.g. chlorophyll-*a*/*b* binding and phycobilisome linker genes; supplementary table S2, Supplementary Material online). In the diatom, *Phaeodactylum tricornutum*, 2 members of the *CRY* photolyase family (*CFP1*, and *CRYP*) participate in the light-dependent expression of photosynthetic light-harvesting genes and photoprotection (Coesel et al. 2009; Juhas et al. 2014; Taddei et al. 2016; Allorent and Petroutsos 2017). It is well-established that *pCRY*s stimulate transcription of nuclear-encoded plastid-targeted proteins in diatoms and land plants (Thum et al. 2001; Ichikawa et al. 2004; Noordally et al. 2013; Fortunato et al. 2015). Using a heterologous expression system (*A*. *thaliana*), the product of the *Gc-pCRY2* gene in *G*. *chorda* was nuclear localized (Fig. 2). These results suggest that the expression pattern of *Gc-pCRY2* gene is also potentially involved in regulating nuclear-encoded plastid-targeted proteins during the day (Fig. 2, supplementary table S2, Supplementary Material online).

The *GcDASH-CRY1*, *GcDASH-CRY3*, and *GcDASH-CRY4* genes in *G*. *chorda* show rhythmic expression (Fig. 2). Interestingly, two of them (*GcDASH-CRY1* and *GcDASH-CRY3*) exhibit an out-of-phase gene expression pattern (pattern-h) compared to *Gc-pCRY2* (pattern-b) in *G*. *chorda* (Fig. 2). The product of one of these genes, *GcDASH-CRY3*, shows plastid localization in the heterologous system (Fig. 2). Therefore, we propose that a diurnal rhythm exists for this plastid-targeted *GcDASH-CRY3* gene, with maximum expression in the evening phase. This pattern may be explained by the repression of photosynthetic activity in the dark period, as observed in *Chlamydomonas reinhardtii* CRY-DASH1 (Rredhi et al. 2021). This mechanism may allow photosystem repair, similar to cyanobacterial DASH-CRY (Syn-CRY) that is involved in PSII repair (Vass et al. 2014).

By contrast with the plastid localization of the *GcDASH-CRY3* gene product, the *GcDASH-CRY1* and *GcDASH-CRY4* gene products are localized to the cytoplasm (Fig. 2). Based on their expression patterns, these genes may also function as photoreceptors at dawn. This suggests that darkness may be required to sustain the “dark phase” of expression. Cytoplasmic *DASH-CRY*s in *G*. *chorda* could therefore be involved in rhythmic regulation through diverse protein interactions in the cytoplasm, like other known *CRY* families that play important roles in signal transduction of cellular metabolism (Wu and Spalding 2007), suppression of nuclear-encoded genes (Chaves et al. 2011; Lee et al. 2015), and protein–protein interactions (Yoo et al. 2013; Damulewicz and Mazzotta 2020) in the cytoplasm. Owing to the similar gene expression pattern for plastid-targeted *GcDASH-CRY3* and cytoplasmic *GcDASH-CRY1*, we propose that these proteins function at dawn in *G*. *chorda*. By contrast, gene expression of *GcDASH-CRY2* is nonrhythmic, although it appears to increase when the cells are exposed to light (i.e. the dusk-phase; Fig. 2). Unfortunately, the subcellular localization of this protein is difficult to determine using heterologous expression due to protein aggregation in the cytoplasm (Fig. 2). Hence, the regulatory activity of *GcDASH-CRY2* remains unclear in *G*. *chorda*. Our data suggest that the *DASH-CRY* family may function in both the plastid and cytoplasm which are likely to be involved in coordinating photosynthetic function in the plastid with nuclear gene transcription and regulation of protein functions in the cytoplasm at daybreak and mostly throughout the light phase. In comparison, the nonrhythmic *Gc-aCRY* and the rhythmic HGT-derived *Gc-hCRY* gene products show nuclear localization in the heterologous system (Fig. 2), thus, they too likely regulate nuclear-encoded genes.

### A Role for *COP1* in *pCRY* Signaling in *G*. *chorda*

Although the origin of *PHY*s and *CRY*s remains controversial in eukaryotes, genes for both photoreceptor families were likely present in the common ancestor of Archaeplastida (Asimgil and Kavakli 2012; Duanmu et al. 2014; Rockwell and Lagarias 2017, 2020). Given the lack of *PHY*s in red algae and in their heterotrophic ancestor Rhodelphidia (Rockwell and Lagarias 2020), it is not surprising that their signaling components are reduced in red algal genomes (Han et al. 2019). Surprisingly, most of the *CRY*-interactive components and typical circadian core oscillator genes found in the land plants are absent in Rhodophyta except for the *COP1* gene and *CCA1*/*LHY*-*RVE8* homologous genes (supplementary table S3, Supplementary Material online; De Caluwé et al. 2016; Linde et al. 2017; Wang and Lin 2020).

COP1 is an E3 ubiquitin ligase involved in degradation of transcription factors (e.g. BBX21, HY5, and HYH) in darkness (Xu et al. 2016). In *G*. *chorda*, *COP1* (PXF44200.1), whose expression increases rapidly at dawn and then gradually falls (pattern-l; supplementary table S3, Supplementary Material online). By comparison, *COP1* gene expression in plants is not light regulated. Plant COP1 function is inhibited by photoactivated CRY and is active in darkness to degrade factors that promote photomorphogenesis (Wang et al. 2001; Holtkotte et al. 2017; Kim et al. 2017; Wang and Lin 2020). Assuming a conserved function for *G*. *chorda* plant-type *CRY*s, the rhythmic patterns of *COP1* (PXF44200.1) and *pCRY* (PXF48820.1) expression would lead to an analogous light-dependent suppression of COP1 function in *G*. *chorda*, while also accounting for the increase in *G*. *chorda COP1* gene expression following inactivation of *pCRY* in darkness (Fig. 2; supplementary table S3, Supplementary Material online). In plants, this process is mediated by COP1-interacting “suppressor of PHYA-105” (SPA) proteins (Holtkotte et al. 2017; Wang and Lin 2020; Ponnu and Hoecker 2021), which lacks supporting evidence in *G*. *chorda* (supplementary table S3, Supplementary Material online). These results suggest that the interplay between CRY and COP1 by light signaling and the physiological cycle differs in plants and red algae, but their signaling output may be similar.

Two genes encoding transcription factors (PXF45473.1 and PXF40765.1; BLASTp top hits, *e*-value cutoff = 1.*e*-05) in *G*. *chorda* which have partial sequence similarity to *CCA1*/*LHY* (AT2G46830/AT1G01060) and *RVE8* (AT3G09600) show gene expression peaks at dawn (supplementary fig. S2, Supplementary Material online). CCA1/LHY and RVE8 repress and activate, respectively, the transcription of *FKF1*, a crucial regulator of photoperiodic flowering in *Arabidopsis* (Schaffer et al. 1998, Wang and Tobin 1998; Imaizumi et al 2005; Sawa et al. 2007; Nakamichi 2020; Lopez et al. 2021). The *FKF1* (AT1G68050) homolog (PXF45752.1; BLASTp top hit, *e*-value cutoff = 1.*e*-05) shows a reversed gene expression pattern to the dawn-peaked transcription factors in *G*. *chorda* (supplementary fig. S2, Supplementary Material online). Consequently, we propose that these transcription factors suppress the transcription of the *FKF1* homologs in *G*. *chorda*. In addition, there were no genes homologous to *LNK*s (AT5G64170 and AT3G54500; BLASTp search, *e*-value cutoff = 1.*e*-05) in *G*. *chorda*. LNKs serve as interacting partners of RVE8 to activate *FKF1* in *Arabidopsis* (Rawat et al. 2011; Fogelmark and Troein 2014; Gray et al. 2017; Nakamichi 2020). Therefore, the dawn-peaked transcription factors in *G*. *chorda* appear to function similarly to CCA1/LHY. However, the roles of these transcription factors, their interacting partners, and the associated transcription-translation feedback loops remain unknown in the developmental processes of red seaweeds. A more in-depth study, employing diverse molecular and genetic approaches targeting homologs of *CCA1*/*LHY* and *RVE8* in *G*. *chorda*, will be necessary to address these shortfalls.

### HGT-derived Rhythmic Genes in *G*. *chorda*

HGTs lead to increased genetic and functional diversity in many eukaryotic genomes (Husnik and McCutcheon 2017; Van Etten and Bhattacharya 2020; Pereira et al. 2022). We analyzed rhythmic genes in *G*. *chorda* to identify candidates that originated *via* HGT (supplementary table S4, Supplementary Material online). One such example is the HGT-derived *Gc-hCRY* gene (Fig. 2). Other examples include two 2-isopropylmalate synthase (K01649) genes (PXF49417.1 and PXF49429.1), a tyrosyl-tRNA synthetase (K01866) gene (PXF50084.1), and a chlamydia-derived ABC transporter gene (PXF50084.1) that encodes a *NitT*/*TauT* family transport system permease (K02050). The latter may have been acquired during primary endosymbiosis because this chlamydia-derived gene is found in red algae as well as in the green lineage (supplementary table S4, Supplementary Material online). Whereas species- or lineage-specific gene transfers can provide novel functions related to nutrition, protection, and adaptation to extreme environments, most transferred genes are nonfunctional (Huang et al. 2004; Nowack et al. 2008; Schönknecht et al. 2013; Wybouw et al. 2014; Husnik and McCutcheon 2017; Rossoni et al. 2019; Lhee et al. 2020). We postulate that such HGT-derived genes could be independently and gradually established through diverse evolutionary processes, likely at the population/species-level (e.g. pangenome concept; Fan et al. 2020; Lee 2021) to the lineage-level (e.g. endosymbiotic gene transfer). Most of the prokaryotic HGT-derived genes in *G*. *chorda* have unknown functions (supplementary table S4, Supplementary Material online), therefore their potential role in host rhythmic gene expression remains a goal of future studies.

### C_3_ Cycle, and Glycolysis/Gluconeogenesis in *G*. *chorda*

The C_3_ (Calvin–Benson–Bassham) cycle corresponds to the light-independent chemical reaction of photosynthesis that fixes carbon dioxide *via* reductive generation of glyceraldehyde-3-phosphate (G3P). The C_3_ cycle consists of three phases: (i) carbon fixation, (ii) reduction, and (iii) regeneration (Gurrieri et al. 2021). During carbon fixation, ribulose-1,5-bisphosphate carboxylase-oxygenase (RuBisCo) catalyzes carbon dioxide-dependent carboxylation of ribulose-1,5-bisphosphate (RuBP) to generate two molecules of glycerate-3-phosphate (3-PGA). Transcription of *RuBisCo* subunits in *G*. *chorda* increased during the light phase, and decreased during the dark phase (supplementary fig. S3, Supplementary Material online). Except nonrhythmic RuBisCo, most of the genes involved in the reduction and regeneration phase of the C_3_ cycle followed pattern-a—the major expression pattern of photosynthesis-associated, plastid-targeted enzymes (Fig. 1, supplementary fig. S3, Supplementary Material online).

Such morning-phased genes show a transcriptional peak at or near dawn, having gradually increased during the night (supplementary fig. S3 and table S5, Supplementary Material online). Expression of genes encoding *RuBisCo* subunits could be generally induced by light availability, but these patterns are not consistent (i.e. fluctuating or nonrhythmic) with species-specific patterns throughout the diel cycle (Recuenco-Muñoz et al. 2015; Perdomo et al. 2021). The distinct pattern of *RuBisCo* expression, i.e. gradual increase in the light period and decrease in darkness, likely reflects the fact that both subunits of this enzyme are plastid-encoded in red algae (supplementary fig. S3, Supplementary Material online). This pattern is unlike the case in the green lineage where the small subunit is nuclear-encoded (Lee et al. 2016). In addition, the discordance between transcript and protein abundance of *RuBisCo* subunits has been reported and reflects post-transcriptional regulation (Recuenco-Muñoz et al. 2015; Perdomo et al. 2021). Therefore, in this study, we focused on carbon metabolism downstream of RuBisCo over the diurnal cycle.

Genes for cytosolic carbon storage pathways in *G*. *chorda*, from hexose-phosphates to floridean starch and floridoside, primarily exhibit morning-phased expression, whereas genes for their breakdown for energy metabolism exhibit dusk- to dark phase maxima (supplementary fig. S3, Supplementary Material online). Such patterns of C_3_ cycle and starch synthesis/breakdown-related gene expression mirror those in the green lineage. The cytoplasmic localization of starch synthesis and breakdown-involved genes in red algae is a striking difference from the plastid localization of these processes in the green lineage (Viola et al. 2001; Patron and Keeling 2005; Ball et al. 2011). Presumably, this allows for more rapid mobilization of stored energy reserves, which may be more critical for red algae, and provides a point of divergence for the regulation of these pathways between these two Archaeplastida lineages.

Genes involved in the glycolysis/gluconeogenesis pathways in *G*. *chorda* typically show dusk- to dawn-phased maxima with out-of-phase of those of the C_3_ cycle and light-harvesting genes (supplementary fig. S3 and table S6, Supplementary Material online). Plastidial glycolysis genes exhibit a transcriptional peak at the evening phase (supplementary fig. S3 and table S6, Supplementary Material online). Gluconeogenesis is a key interface between organic acid and sugar metabolism—a process that generates glucose from oxaloacetate (OAA) in plants (Leegood and Walker 2003; Walker et al. 2021). In general, gluconeogenesis in plants is dependent on two types of pathways that generate PEP molecules. Key enzymes in this pathway are pyruvate orthophosphate dikinase (PPDK; pyruvate → PEP), and PEP carboxykinase [PEPCK; OAA → PEP + CO_2_] (Walker et al. 2021). In red algae, PEPCK (EC 4.1.1.49, K01610) is restricted to Cyanidiophyceae, Bangiophyceae, and Compsopogonophyceae (supplementary table S7, Supplementary Material online). Therefore, gluconeogenesis in most (ca. 98%; Oct. 2022; https://www.algaebase.org) red algal species (i.e. Florideophyceae including *G*. *chorda*) is dependent primarily on PPDK (supplementary fig. S3 and table S7, Supplementary Material online). PEP molecules for gluconeogenesis could be primarily produced from OAA *via* cytosolic malate and pyruvate metabolism in red algae.

### Regulation of Cytosolic and Mitochondrial Carbon Metabolism in *G*. *chorda*

**Fig. 3.**
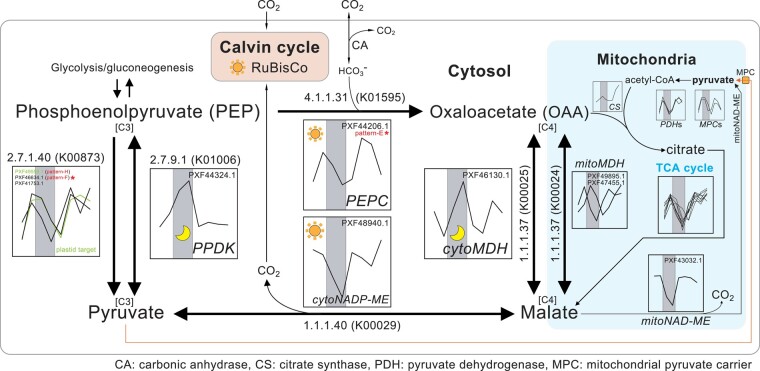
Model for the DN cycle of gene expression involving metabolism of cytosolic C_3_ and C_4_ organic acids in *G*. *chorda* (asterisk: significant correlation in rhythmic pattern).

*PEPC* is highly expressed at the dusk-phase in *G*. *chorda* (pattern-e; Fig. 3), and likely plays an important role in the induction of cytosolic OAA (Shi et al. 2015; Walker et al. 2021; Shu et al. 2022). This enzyme is supported by carbonic anhydrase (CA) which catalyzes the interconversion of carbon dioxide to bicarbonate (CO_2_ + H_2_O ←→ HCO_3_  ^−^ + H^+^)—a major CO_2_-concentrating mechanism (CCM) in *G*. *chorda* (Fig. 3, supplementary table S8, Supplementary Material online; Badger and Price 1994; Lindskog 1997). The pattern of gene expression in *G*. *chorda* is consistent with primary carbon fixation by C_3_ cycle morning-phased genes in plastids followed by secondary carbon fixation in the cytosol with PEPC at dusk and in the early evening. For sequential carbon fixation to function, proper regulation of the cytosolic CO_2_ and/or bicarbonate ions (HCO_3_  ^−^) concentrations is important and both cytosolic nicotinamide adenine dinucleotide phosphate-malic enzyme (cytoNADP-ME) and mitochondrial nicotinamide adenine dinucleotide-malic enzyme (mitoNAD-ME) are critical for both types of carbon fixation. Whereas cytoNADP-ME performs nonphotosynthetic functions (e.g. stress responses, cytosolic pH regulation, and TCA cycle metabolism) in C_3_ and C_4_ plants, cytoNADP-ME is utilized by CAM plants for CO_2_ release in a process analogous to that performed by the plastid NADP-ME in C_4_ plants. Both reactions produce CO_2_ which is fixed by the C_3_ cycle enzyme RuBisCo (Drincovich et al. 2001; Lai et al. 2002; Wheeler et al. 2005; Maier et al. 2011; Tronconi et al. 2018; Chen et al. 2019). We postulate that cytosolic CO_2_ produced by cytoNADP-ME supports carbon fixation in both the C_3_ cycle (morning) and the CCM (dusk) because cytosolic CO_2_ during daytime can easily diffuse into plastids. In addition, mitoNAD-ME can elevate cellular CO_2_ concentration (Fig. 3). Even though the major function of mitoNAD-ME is in the TCA cycle, CO_2_ diffusion likely contributes to plastid CO_2_ fixation in both C_4_ and CAM plants (Hatch and Kagawa 1974; Artus and Edwards 1985; Tronconi et al. 2018) and, by analogy in *G*. *chorda*. In the C_3_  *A*. *thaliana*, however, *mitoNAD-ME* genes are highly active during night (Tronconi et al. 2008, 2018). Therefore, the daytime reaction of mitoNAD-ME in *G*. *chorda* could induce CO_2_ diffusion into the cytosol, and this contributes to both types (RuBisCo and PEPC) of light-activated carbon fixation like cytoNAD-ME (Fig. 3, supplementary fig. S3, Supplementary Material online). The TCA cycle (active at dusk; supplementary fig. S4, Supplementary Material online) also generates CO_2_ thus it could be simultaneously recycled by PEPC in the cytosol.

**Fig. 4.**
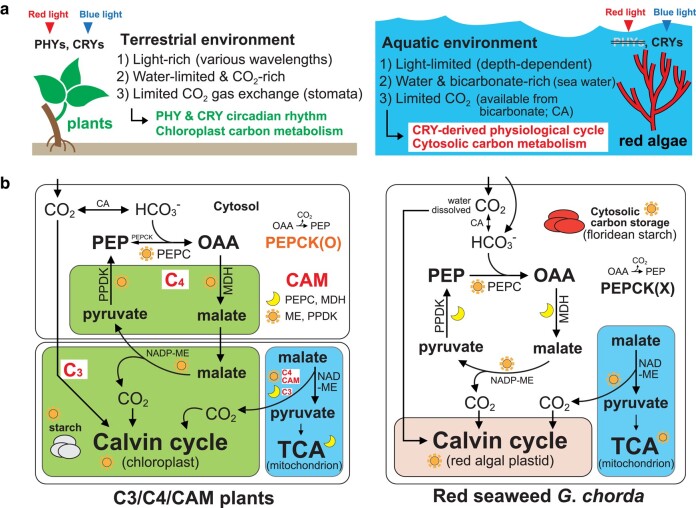
Comparison of diurnal gene regulation and cellular metabolism in plants and red algae. a) Comparison of major DN regulatory systems in terrestrial and aquatic habitats. b) Comparison of carbon metabolism in plants and *G*. *chorda*. The simplified model of chloroplast carbon metabolism in C_3_, C_4_, and CAM plants is based on previous studies (Drincovich et al. 2001; Lai et al. 2002; Wheeler et al. 2005; Tronconi et al. 2008, 2018; Aubry et al. 2011; Maier et al. 2011; Zones et al. 2015; Rao and Dixon 2016; Shen et al. 2017; Chen et al. 2019; Khoshravesh et al. 2020; Tay et al. 2021; Winter and Smith 2022), whereas the model of cytosolic carbon metabolism in *G*. *chorda* is based on gene expression analysis (this study). Abbreviations: PHY, phytochrome; CRY, cryptochrome; CA, carbonic anhydrase; PEP, phosphoenolpyruvate; OAA, oxaloacetate; PEPC, PEP carboxylase; PEPCK, PEP carboxykinase; MDH, malate dehydrogenase; PPDK, pyruvate orthophosphate dikinase; NADP-ME, NADP-malic enzyme; NAD-ME, NAD-malic enzyme; Calvin C_3_ cycle, Calvin–Benson–Bassham cycle; TCA cycle, tricarboxylic acid cycle.

The loss of *PEPCK* in red algae may indicate that it is unnecessary to supply additional CO_2_ for photosynthetic carbon fixation as in C_4_ plants, because of the bicarbonate-rich condition (about 90% of inorganic carbon sources) in most marine environments (Poschenrieder et al. 2018), which could be captured by PEPC. Moreover, the addition of sodium bicarbonate promotes growth in red seaweeds and photosynthetic activity (Zhou et al. 2016). In contrast, the availability of water and HCO_3_  ^−^ in land plants is frequently limiting in terrestrial environments. In addition, CO_2_ availability could be limited by closed stomata in land plants under high temperatures and drought (Poschenrieder et al. 2018), thus land plants may have evolved enhanced photosynthetic carbon fixation in chloroplasts to inhibit photorespiration: e.g. C_4_-type photosynthesis (Sage et al. 2012). Photorespiration in aquatic algae could also occur under CO_2_ deficiency but HCO_3_  ^−^ availability by CA reduces photorespiration (Li et al. 2021). Although cytosolic PEPC is present both in red algae and land plants, the differences in availability of CO_2_ and HCO_3_  ^−^ could lead to divergent strategies for carbon metabolism. Algal species use inorganic carbon transport (not stomata), thus inorganic carbon is more accessible to these taxa (Raven and Beardall 2016; DiMario et al. 2017; Razzak et al. 2018). Aquatic green algae are similar, but they have chloroplast-centralized carbon metabolism, and energy metabolism is active under dark conditions, as in land plants (Zones et al. 2015). Therefore, we suggest that metabolic processes and cellular structures underpinning carbon metabolism in red algae and the green lineages have diverged significantly (Fig. 4).

Based on these results, we postulate that ancient carbon metabolism in the major red algal lineage Florideophyceae evolved independently as a compact (e.g. *CRY*s) and efficient system. This is in contrast to the spatial (pyruvate and malate synthesis in mesophylls and bundle sheath in C_4_ plants) or temporal (carbon fixation at the daytime and dark energy metabolism in most C_3_/C_4_/CAM plants) gap to enhance photosynthesis in land plants. The evolutionary trends in carbon metabolism in red algae are quite different from the systems in C_3_, C_4_, or CAM plants.

### Comparative Models of Photoreceptors and Carbon Metabolism Between Land Plants and Red Seaweeds

The central input pathways of circadian oscillation are controlled by light cues primarily mediated by the photoreceptors PHY and CRY in green plants (Lopez et al. 2021; Liu et al. 2022). PHYs are absent in red algae (Duanmu et al. 2014), therefore, CRYs, that are responsive to blue light provide the primary input to the oscillatory mechanism. These light wavelengths penetrate more deeply in water than does red light. For this reason, the CRY-based input system appears to be sufficient for *G*. *chorda* to flourish in blue light-enriched subtidal marine environments that have (depth-dependent) lower incident light levels. By contrast, green algae and plants are better adapted to shallower aquatic and terrestrial environments, where red wavelengths are more abundant (Fig. 4a), accounting for their retention of PHY sensors. For these reasons, the role of light sensing in driving physiological cycles in red algae is likely to differ significantly from that found in the green lineage.

Given this hypothesis, we did RNA-seq experiments to identify rhythmic genes and the role of light in *G*. *chorda* biology. Quantitative real-time PCR (qRT-PCR) analysis of gene expression patterns for most of the target genes provides results that are consistent with the RNA-seq data (See Methods). Based on the RNA-seq results, we identified 3,098 rhythmic genes in this red seaweed (Fig. 1, supplementary table S1, Supplementary Material online). Among genes of particular interest, were those involved in light sensing and photosynthesis. We detected rhythmic gene expression patterns in 5/8 *CRY*s (i.e. one *pCRY*, three *DASH-CRY*s, and one HGT-derived *CRY*-like) and many genes involved in photosynthetic/cytosolic carbon metabolism (Figs. 2 and 3, supplementary fig. S3 and table S2, Supplementary Material online). PHY- and CRY-based modulation of the oscillatory mechanism in the Archaeplastida evolved independently of light-mediated regulation of the *kai* family-based clock found in the cyanobacterial ancestor of plastids (Kondo and Ishiura 2000). Upon the loss of *PHY*s in red algae, rhythmic gene expression of *pCRY* and *DASH-CRY*s became associated with photosynthesis. The organization of these pathways is however likely to fundamentally differ from that in green lineage because highly conserved PHY- and CRY-interacting factors in plant species are absent in *G*. *chorda* (supplementary table S3, Supplementary Material online). Therefore, the diurnal cycle and its role in red algal physiology also diverged in a lineage-specific manner through processes such as gene loss, gene family expansion, and HGT (supplementary table S4, Supplementary Material online).

The green algae and most land plants retain the ancestral form of carbon metabolism, i.e. the C_3_ pathway, present in cyanobacteria (Facchinelli and Weber 2011). However, some land plant lineages have independently evolved specialized forms of photosynthetic carbon metabolism (e.g. C_4_, and CAM) as an adaptation to CO_2_ limitation (Fig. 4a). Mesophyll and bundle sheath cells allow C_4_ plants to overcome CO_2_ gas exchange limitation and reduce the rate of photorespiration (Wang et al. 2014). In marine environments, HCO_3_  ^−^ is more abundant than dissolved CO_2_, owing to the high pH (∼ pH 8.1) of seawater (Fig. 4a). For this reason, CAs perform essential roles in marine species, not only in the interconversion of carbon sources (CO_2_ + H_2_O ←→ HCO_3_  ^−^ + H^+^) for photosynthesis, but also in diverse forms of carbon metabolism (e.g. balancing cellular pH levels; DiMario et al. 2017).

We propose that in *G*. *chorda*, HCO_3_  ^−^ metabolism occurs *via* rhythmic expression of *PEPC* without *PEPCK* (Fig. 4b). Based on transcriptional profiling of *G*. *chorda*, we suggest that red seaweeds contain a more compact (i.e. reduced) mechanism for carbon metabolism than do green lineages, based on the following observations (Fig. 4b): (i) photosynthetic carbon fixation and cytosolic storage (i.e. floridean starch) in *G*. *chorda* are primarily induced at morning, whereas their breakdown (e.g. starch degradation and glycolysis) exhibit dusk- to dark phase maxima. By contrast with C_3_ plants, the daytime supply of CO_2_ in *G*. *chorda* relies on malic enzymes (MEs) that feed photosynthetic carbon fixation and reduce photorespiration. (ii) HCO_3_  ^−^ (and CO_2_  *via* carbonic anhydrases, including remnants from photosynthetic carbon fixation) is captured by PEPC (rhythmic gene expression) at dusk (supplementary fig. S3, Supplementary Material online). The synthesis of OAA by PEPC could directly feed the TCA cycle in red algae during the dusk-phase. (iii) Pyruvate kinases (PEP → pyruvate) and the TCA cycle are also active at dusk, therefore carbon flow from C_3_ organic acids (i.e. PEP) to energy metabolism in *G*. *chorda* is directly associated during the daytime, whereas energy metabolism in the green lineages is active in the dark (Figs. 3 and 4b). In addition, cytosolic CO_2_ diffusion from the TCA cycle (dusk) and NAD-ME (daytime) could be recycled by photosynthetic (C_3_ cycle in the morning) and cytosolic (PEPC at dusk) carbon fixation. Dusk activation of genes involved in mitochondrial pyruvate metabolism (e.g. pyruvate dehydrogenases and mitochondrial pyruvate carriers) and oxidative phosphorylation, including the F-type (mitochondria/chloroplast) ATPases (Kühlbrandt 2019), also support increased energy metabolism at dusk in *G*. *chorda* (Fig. 3, supplementary fig. S5, Supplementary Material online). Therefore, *G*. *chorda* exhibits a compact cytosolic carbon metabolism, whereby photosynthetic carbon fixation and energy metabolism are tightly coupled in daylight (Fig. 4b). (iv) One-way gluconeogenesis (from OAA to glucose) in *G*. *chorda* is controlled by the day (PEPC/NADP-ME) and night (PPDK/MDH) cycle, which is closely related to efficient carbon re-cycling in *G*. *chorda* (Fig. 3).

## Conclusion

The green lineage, in particular land plants, evolved a system to balance water loss and CO_2_ capture in terrestrial environments using spatial or temporal isolation of pyruvate and malate *via* the C_4_ or CAM system, respectively. In contrast, red algae maintain a reduced set of photoreceptors and a compact cytosolic carbon metabolism to exploit limited light and carbon (i.e. ∼10,000 times lower CO_2_ gas diffusion rate than in air; Raven 1970; Zeebe 2011) conditions in aquatic environments (Fig. 4b). We note that our results and hypotheses regarding diverse enzymatic processes in red seaweeds are based solely on transcriptome analysis. Given that post-transcriptional regulation is common in eukaryotes and often unlinks gene expression from protein accumulation (Fang et al. 1998; Yu 2007; Yu et al. 2021), our data need to be interpreted with some caution. In addition, the putative functions of rhythmic genes and the encoded proteins we have identified are based on inferences using other model systems and are therefore provisional in nature. Nevertheless, our results provide novel insights into the regulation of physiological cycles that can be tested using other omics and genetic approaches. In addition, industrial applications using carbon metabolism in the red seaweeds (e.g. CO_2_ hydration; Razzak et al. 2020) may be used to mitigate carbon emissions. More generally, our study suggests that gaining a deeper understanding of red algal carbon metabolism may contribute to the understanding of ecological carbon fluxes and storage in marine ecosystems (e.g. blue carbon; Krause-Jensen et al. 2018; Macreadie et al. 2019).

## Materials and Methods

### Preparation of Algal Samples and RNA Sequencing

The thallus of *G*. *chorda* was collected from an aquaculture farm at Jangheung (South Korea) and washed several times with sterile seawater to remove surface contaminants. The seaweed was cut into 10 cm fragments, pre-cultured in L1 medium (L1 media kit, https://ncma.bigelow.org/MKL150L) for two weeks. Algal cultures were maintained at 15 °C under a 12L/12D photoperiod (12:12 h light and dark cycle) with 1,500 lux light intensity. To examine rhythmic gene expression in *G*. *chorda*, algal samples were exposed to a DN cycle for 24 h (12 h-light:12 h-dark) and then CL for 24 h. We collected samples after 4 h-light exposure (DN4) initially to analyze morning-phased gene expression, and then each 6 h in DN (DN10, DN16, and DN22) and CL conditions (CL4, CL10, CL16, and CL22). For example, DN10 and DN16 designated the experimental condition of 10 h-light exposure and 12 h-light/4 h-dark exposure, respectively. CL4 indicated 4 h-CL exposure after the DN period for 24 h. Algal samples were prepared in triplicate (except for one condition; we used duplicate sequencing data of CL16 because of failed sequence library construction) and stored at −80 °C prior to RNA extraction.

Total RNA was extracted from 3 different independent samples in each condition using the RNeasy Plant Mini Kit (QIAGEN, Germany). The quality of purified RNAs was determined using a 2100 Bioanalyzer (Agilent Technologies, USA). RNA sequencing libraries were constructed using the TruSeq RNA Sample Prep Kit (Illumina, USA). Libraries were sequenced using 100 bp paired-end reagents on the Illumina HiSeq2500 platform (Illumina, USA). All experiments were done using the manufacturer's instructions. The quality scores of RNA-seq reads (average Q20 = 97% and Q30 = 92%) were analyzed using Fastp (v0.23.4; default options; Chen et al. 2018; Chen 2023).

### Analysis of Rhythmic Gene Expression Patterns in *Gracilariopsis chorda*

The RNA-seq reads (NCBI SRA database; SRR21594546—SRR21594568; BioProject PRJNA872288) of *G*. *chorda* were trimmed using Trimmomatic (v0.39; default options; Bolger et al. 2014) and mapped to coding sequences (NCBI genome accession NBIV00000000.1; Lee et al. 2018) from this species using Salmon (v1.4.0; default options; Patro et al. 2017). Based on the transcripts per million (TPM) value of each gene, those with low TPM values (<0.1) were removed. Organelle genes were analyzed with ChloroSeq using the reads per kilobase million (RPKM) values (filtration cutoff: < 0.1), which is an optimized organelle RNA-seq bioinformatic pipeline including the Tophat2 aligner (Kim et al. 2013; Smith and Lima 2017). After the filtration step, a total of 7,985 genes were selected for analysis of gene expression. To validate the gene expression results in triplicate (or duplicate) RNA-seq data for each condition, we conducted Pearson correlation analysis (pearsonr; scipy.stats python module v0.13.0b1; python v2.7.16). The average correlation coefficient values for each condition ranged from 0.93 to 0.99, with *P*-value < 0.05. These results indicate that the observed gene expression patterns under each experimental condition are consistent and the observed variation is not statistically significant. To identify rhythmic gene expression, we generated average TPM or RPKM values for each condition, and did 3 types of comparative analyses based on the z-scores ([“TPM value”—“average TPM values of all conditions in each gene”]/“Standard deviation of all conditions in each gene”) between the 24 h-DN period (12 h-light and 12 h-dark; DN4, DN10, DN16, and DN22) and the latter period (24 h-light; CL4, CL10, CL16, and CL22).

The first approach was a selection of shared gene expression patterns with respect to the time between the two periods (i.e. “DN4—DN22” and “CL4—CL22”). We used the relative gene expression patterns (i.e. z-scores) with “up (+)” and “down (−)” indicating “increase” and “decrease” of gene expression compared to the previous time point, respectively (supplementary fig. S6, Supplementary Material online). For example, z-scores of “PXF48091.1” were 1.71 (DN4), −0.94 (DN10), −0.49 (DN16), 0.41 (DN22), 0.92 (CL4), −1.16 (CL10), −0.67 (CL16), and 0.23 (CL22) that indicated a “down-up-up/up/down-up-up” pattern. The same patterns occurred between the 2 periods (i.e. “down/up/up”) but we did not consider the transition point between DN22 and CL4 because it is not directly related to the rhythmic pattern at this stage. Based on the up and down gene expression patterns, a total of 3,098 rhythmic gene expressions were selected (the green asterisk in supplementary fig. S6, Supplementary Material online). The second approach was analysis of periodicity in each gene expression pattern. To this end, we used MetaCycle (meta2d; Wu et al. 2016) and BioCycle (Agostinelli et al. 2016) based on the DN period for 24 h (12 h-light:12 h-dark; DN4, DN10, DN16, and DN22). MetaCycle (Wu et al. 2016) analyzes periodic genes using three methods: ARSER (ARS), JTK_CYCLE (JTK), and Lomb-Scargle (LS). To implement MetaCycle, timepoints with an equal number of biological replicates are required, thus we selected 2 RNA-seq libraries from the existing triplicate datasets because 1 condition (CL16) had only 2 libraries available. For the data collection, the most correlated (duplicate) RNA-seq data from each sample were selected based on Pearson correlation analysis (the highest correlation coefficient with *P*-value < 0.05; pearsonr; scipy.stats python module v0.13.0b1; python v2.7.16). From the 3 types of MetaCycle results (ARS, JTK, and LS), we identified 1,056 periodic genes that show a *P*-value < 0.05 under all algorithms. BioCycle (Agostinelli et al. 2016) is a deep learning method used to recognize periodic genes. We identified 2,370 periodic genes with *P*-value < 0.05 and combined these with the MetaCycle results into an initial candidate list of 2,518 rhythmic genes (the blue asterisk in supplementary fig. S6, Supplementary Material online). However, among the selected rhythmic genes, we excluded 971 genes because they show low sensitivities, which indicates different gene expression patterns between the two periods (e.g. up/up/down and up/down/down in PXF48668.1; supplementary fig. S6, Supplementary Material online), although we used several statistical tests supported by MetaCycle and BioCycle. Therefore, we selected 1,547 genes with high sensitivities as rhythmic genes, which show the same gene expression pattern between the 2 periods with statistical supports as described above (*P*-value < 0.05; MetaCycle and BioCycle). As an additional validation step, we identified 670 genes that have a *P*-value < 0.05 in the Pearson correlation analysis (pearsonr; scipy.stats python module v0.13.0b1; python v2.7.16) of gene expression patterns between the two periods among the 1,547 rhythmic genes in *G*. *chorda*. These genes are marked as “significant correlation” (supplementary table S1, Supplementary Material online). We defined the remaining 1,551 rhythmic gene candidates as “general-rhythm” genes due to the same expression patterns between “DN4—DN22” and “CL4—CL22” (supplementary fig. S6 and table S1, Supplementary Material online). To create graphs of gene expression patterns with the normalized values (z-score), the “matplotlib.pyplot” (v1.3.1), we used a module in Python (v2.7.16).

Rhythmic gene expression patterns were divided into 12 major and two minor patterns that show fluctuating gene expression (Fig. 1). The up- and down-regulated genes from DN4 are shown with “+” and “−”, respectively, which indicate relative gene expression relative to the previous time point. Based on these results, we defined morning-phased (a-b-c; DN4), dusk-phased (d-e-f; DN10), evening-phased (g-h-i; DN16), and dawn-phased (j-k-l; DN22) rhythmic genes (Fig. 1). Within each group, however, up-regulation of gene expression starts at different times. For example, the up-regulation of gene expressions in pattern-a starts at DN10, which is earlier than the others (i.e. pattern-b at DN16, and pattern-c at DN22) within this group (Fig. 1). To infer the *CRY* family data in *G*. *chorda*, we relied on published data (Kianianmomeni and Hallmann 2014).

Functional annotation of protein-coding genes was done using metabolic pathway analysis with the Kyoto Encyclopedia of Genes and Genomes database (http://www.genome.jp/tools/blast/), eggNOG-mapper (Huerta-Cepas et al. 2017), and Conserved Domain (CD) searches (Marchler-Bauer et al. 2017). Predictions of subcellular localization using target proteins were made using ChloroP (v1.1), and TargetP (v1.1) (Nielsen et al. 1997; Emanuelsson et al. 1999, 2000).

### Quantitative Real-time PCR Experiments

To verify the reliability of gene expression patterns in the RNA-seq data, we conducted qRT-PCR experiments. All total RNA samples were pretreated with RNase-free DNase I (Ambion, USA) to eliminate genomic DNA contamination before cDNA synthesis. Reverse transcription of RNA samples was done using the RevertAid First Strand cDNA Synthesis Kit (Fermentas, Lithuania) according to the manufacturer's instructions, and the resulting cDNA products were diluted 1:20 with nuclease-free water. Specific PCR primer pairs for the target genes were designed (supplementary table S9, Supplementary Material online). The qRT-PCR was conducted on a CFX connect Real-Time System (Bio-Rad, Germany) using SsoFast EvaGreen Supermix (Bio-Rad, Germany) in a 10 μL reaction. Preliminary quantitative RT-PCR assays and melting curve analyses were performed to ensure that the primer pairs could efficiently amplify a single product without genomic DNA contamination. Based on the preliminary test, we selected two housekeeping genes in *G*. *chorda*, eukaryotic translation initiation factor 4E (*eIF4E*) and eukaryotic peptide chain release factor subunit 1 (*ERF1*), which showed the least variation in gene expression in our experiments. The qRT-PCR experiments for the target genes were performed with biological triplicates. The relative quantification of each gene expression among samples was evaluated using the comparative ΔΔC_T_ method provided by Bio-Rad program (Bio-Rad CFX Manager 3.1) with two internal controls. To assess the quality of the qRT-PCR experiments, we calculate relative gene expression with error bars (standard error of the mean; supplementary fig. S7, Supplementary Material online).

### Homologous Gene Search and Phylogenetic Analysis

Homologs of target genes were identified using Protein Basic Local Alignment Search Tool (BLASTp) search (top 500–1,000 hits; *e*-value cutoff = 1.*e*-05), and aligned with MAFFT (default option: –auto; v7.487; Yamadat et al. 2016). Available red algal EST (expression sequencing tags) data were collected from the MMETSP database (Keeling et al. 2014; Johnson et al. 2019). Phylogenetic analysis using the alignments was done using maximum likelihood (ML), with IQ-tree v1.6.12 (Nguyen et al. 2015). The ML trees were constructed using model test (-m TEST), and ultrafast bootstrapping of 1,000 replications (-bb 1000). When we constructed a combined alignment (e.g. *CRY*s) including gene families, we collected the top 5 or 10 hits in each taxonomic group from each blast result, and these were combined. The combined protein dataset was aligned using MAFFT (v7.487; Yamada et al. 2016) under default settings and used for phylogenetic analysis.

### Experimental Procedure for Subcellular Localizations

Full-length cDNA fragments lacking a stop codon that encoded the seven *CRY* isoforms in *G*. *chorda* were amplified by PCR with their specific primer pairs (supplementary table S10, Supplementary Material online). PCR products were digested with *Xba*I/*Spe*I and *Bam*HI/*Bgl*II, and ligated in-frame upstream of the green fluorescence protein (*GFP*) gene in the vector 326-sGFP. The *Gc-pCRY1* (PXF43553.1) gene was not used in this study because we were unable to construct the in-frame GFP fusion. To analyze subcellular localizations, *A*. *thaliana* leaf protoplasts were isolated according to Yoo et al. (2007). The in-frame GFP fusion constructs were introduced into *A*. *thaliana* protoplasts with the polyethylene glycol-mediated method (Abel and Theologis 1994), and then incubated for 12–16 h under dark conditions. Images of GFP fluorescence and red chlorophyll autofluorescence in the transformed protoplasts were taken by a cooled charge-coupled device camera and an Olympus BX53 Light/Fluorescence microscope at 40× magnification. We used the fluorescence filter sets of eGFP (Ex: BP470/40, 495DC, Em: BP525/50), and Cy5 (Ex: BP620/60, 660DC, Em: BP700/75) for GFP and chlorophyll autofluorescence, respectively.

### Summary of Carbon Metabolisms in Green Algae and C_3_/C_4_/CAM Plants

A simplified model of carbon metabolism in C_3_/C_4_/CAM plants was generated based on insights gained from a wide variety of studies (Drincovich et al. 2001; Lai et al. 2002; Wheeler et al. 2005; Aubry et al. 2011; Maier et al. 2011; Tronconi et al. 2008, 2018; Zones et al. 2015; Rao and Dixon 2016; Shen et al. 2017; Chen et al. 2019; Khoshravesh et al. 2020; Tay et al. 2021; Winter and Smith 2022).

## Supplementary Material

msae012_Supplementary_Data

## Data Availability

Raw reads of RNA sequencing data in this study were deposited to the NCBI Sequence Read Archive (SRA) database with accession numbers SRR21594546—SRR21594568 (BioProject PRJNA872288).

## References

[msae012-B1] Abel S, Theologis A. Transient transformation of *Arabidopsis* leaf protoplasts: a versatile experimental system to study gene expression. Plant J. 1994:5(3):421–427. 10.1111/j.1365-313X.1994.00421.x. 8180625

[msae012-B2] Agostinelli F, Ceglia N, Shahbaba B, Sassone-Corsi P, Baldi P. What time is it? Deep learning approaches for circadian rhythms. Bioinformatics. 2016:32(12):i8–i17. 10.1093/bioinformatics/btw243. 27307647 PMC4908327

[msae012-B3] Allorent G, Petroutsos D. Photoreceptor-dependent regulation of photoprotection. Curr Opin Plant Biol. 2017:37:102–108. 10.1016/j.pbi.2017.03.016. 28472717

[msae012-B4] Artus NN, Edwards GE. Properties of leaf NAD-malic enzyme from the inducible crassulacean acid metabolism species *Mesembryanthemum crystallinum*. Plant Cell Physiol. 1985:26(2):341–350. 10.1093/oxfordjournals.pcp.a076915

[msae012-B5] Ashbrook LH, Krystal AD, Fu YH, Ptáček LJ. Genetics of the human circadian clock and sleep homeostat. Neuropsychopharmacol. 2020:45(1):45–54. 10.1038/s41386-019-0476-7. PMC6879540 31400754

[msae012-B6] Asimgil H, Kavakli IH. Purification and characterization of five members of photolyase/cryptochrome family from *Cyanidioschyzon merolae*. Plant Sci. 2012:185-186:190–198. 10.1016/j.plantsci.2011.10.005. 22325881

[msae012-B7] Aubry S, Brown NJ, Hibberd JM. The role of proteins in C3 plants prior to their recruitment into the C4 pathway. J Exp Bot. 2011:62(9):3049–3059. 10.1093/jxb/err012. 21321052

[msae012-B8] Badger MR, Price GD. The role of carbonic anhydrase in photosynthesis. Annu Rev Plant Physiol. 1994:45(1):369–392. 10.1146/annurev.pp.45.060194.002101.

[msae012-B9] Ball S, Colleoni C, Cenci U, Raj JN, Tirtiaux C. The evolution of glycogen and starch metabolism in eukaryotes gives molecular clues to understand the establishment of plastid endosymbiosis. J Exp Bot. 2011:62(6):1775–1801. 10.1093/jxb/erq411. 21220783

[msae012-B10] Bhattacharya D, Yoon HS, Hackett JD. Photosynthetic eukaryotes unite: endosymbiosis connects the dots. BioEssays. 2004:26(1):50–60. 10.1002/bies.10376. 14696040

[msae012-B11] Bolger AM, Lohse M, Usadel B. Trimmomatic: a flexible trimmer for illumina sequence data. Bioinformatics. 2014:30(15):2114–2120. 10.1093/bioinformatics/btu170. 24695404 PMC4103590

[msae012-B12] Brown SA, Kowalska E, Dallmann R. (Re)inventing the circadian feedback loop. Dev Cell. 2012:22(3):477–487. 10.1016/j.devcel.2012.02.007. 22421040

[msae012-B13] Brudler R, Hitomi K, Daiyasu H, Toh H, Kucho K, Ishiura M, Kanehisa M, Roberts VA, Todo T, Tainer JA, et al Identification of a new cryptochrome class: structure, function, and evolution. Mol Cell. 2003:11(1):59–67. 10.1016/S1097-2765(03)00008-X. 12535521

[msae012-B14] Castrillo M, García-Martínez J, Avalos J. Light-dependent functions of the *Fusarium fujokuroi* CryD DASH cryptochrome in development and secondary metabolism. Appl Environ Microbiol. 2013:79(8):2777–2788. 10.1128/AEM.03110-12. 23417004 PMC3623198

[msae012-B15] Chaves I, Pokorny R, Byrdin M, Hoang N, Ritz T, Brettel K, Essen LO, van der Horst GTJ, Batschauer A, Ahmad M. The cryptochromes: blue light photoreceptors in plants and animals. Annu Rev Plant Biol. 2011:62(1):335–364. 10.1146/annurev-arplant-042110-103759. 21526969

[msae012-B16] Chen S . Ultrafast one-pass FASTQ data preprocessing, quality control, and deduplication using fastp. iMeta. 2023:2(2):e107. 10.1002/imt2.107. PMC10989850 38868435

[msae012-B17] Chen Q, Wang B, Ding H, Zhang J, Li S. Review: the role of. NADP-malic enzyme in plants under stress. Plant Sci. 2019:281:206–212. 10.1016/j.plantsci.2019.01.010. 30824053

[msae012-B18] Chen S, Zhou Y, Chen Y, Gu J. Fastp: an ultra-fast all-in-one FASTQ preprocessor. Bioinformatics. 2018:34(17):i884–i890. 10.1093/bioinformatics/bty560. 30423086 PMC6129281

[msae012-B19] Chollet R, Vidal J, O’Leary MH. Phosphoenolpyruvate carboxylase: a ubiquitous, highly regulated enzyme in plants. Annu Rev Plant Physiol. 1996:47(1):273–298. 10.1146/annurev.arplant.47.1.273. 15012290

[msae012-B20] Coesel S, Mangogna M, Ishikawa T, Heijde M, Rogato A, Finazzi G, Todo T, Bowler C, Falciatore A. Diatom PtCPF1 is a new cryptochrome/photolyase family member with DNA repair and transcription regulation activity. EMBO Rep. 2009:10(6):655–661. 10.1038/embor.2009.59. 19424294 PMC2711838

[msae012-B21] Corellou F, Schwartz C, Motta J-P, Djouani-Tahri EB, Sanchez F, Bouget F-Y. Clocks in the green lineage: comparative functional analysis of the circadian architecture of the picoeukaryote *Ostreococcus*. Plant Cell. 2009:21(11):3436–3449. 10.1105/tpc.109.068825. 19948792 PMC2798331

[msae012-B22] Damulewicz M, Mazzotta GM. One actor, multiple roles: the performances of cryprtochrome in *Drosophila*. Front Physiol. 2020:11:99. 10.3389/fphys.2020.00099. 32194430 PMC7066326

[msae012-B23] De Caluwé J, Xiao Q, Hermans C, Verbruggen N, Leloup JC, Gonze D. A compact model for the complex plant circadian clock. Front Plant Sci. 2016:7:74. 10.3389/fpls.2016.00074. 26904049 PMC4742534

[msae012-B24] de Dios VR, Gessler A. Circadian regulation of photosynthesis and transpiration from genes to ecosystems. Environ Exp Bot. 2018:152:37–48. 10.1016/j.envexpbot.2017.09.010.

[msae012-B25] de Goede P, Wefers J, Brombacher EC, Schrauwen P, Kalsbeek A. Circadian rhythms in mitochondrial respiration. J Mol Endocrinol. 2018:60(3):R155–R130. 10.1530/JME-17-0196. PMC5854864 29378772

[msae012-B26] DiMario RJ, Clayton H, Mukherjee A, Ludwig M, Moroney JV. Plant carbonic anhydrases: structures, locations, evolution, and physiological roles. Mol Plant. 2017:10(1):30–46. 10.1016/j.molp.2016.09.001. 27646307 PMC5226100

[msae012-B27] Dodd AN, Kusakina J, Hall A, Gould PD, Hanaoka M. The circadian regulation of photosynthesis. Photosynth Res. 2013:119(1-2):181–190. 10.1007/s11120-013-9811-8. 23529849

[msae012-B28] Drincovich MF, Casati P, Andreo CS. NADP-malic enzyme from plants: a ubiquitous enzyme involved in different metabolic pathways. FEBS Lett. 2001:490(1-2):1–6. 10.1016/S0014-5793(00)02331-0. 11172800

[msae012-B29] Duanmu D, Bachy C, Sudek S, Wong C-H, Jiménez V, Rockwell NC, Martin SS, Ngan CY, Reistetter EN, van Baren MJ, et al Marine algae and land plants share conserved phytochrome signaling systems. Proc Natl Acad Sci U S A. 2014:111(44):15827–15832. 10.1073/pnas.1416751111. 25267653 PMC4226090

[msae012-B30] Emanuelsson O, Nielsen H, Brunak S, von Heijne G. Predicting subcellular localization of proteins based on their N-terminal amino acid sequence. J Mol Biol. 2000:300(4):1005–1016. 10.1006/jmbi.2000.3903. 10891285

[msae012-B31] Emanuelsson O, Nielsen H, von Heijne G. Chlorop, a neural network-based method for predicting chloroplast transit peptides and their cleavage sites. Protein Sci. 1999:8(5):978–984. 10.1110/ps.8.5.978. 10338008 PMC2144330

[msae012-B32] Facchinelli F, Weber APM. The metabolite transporters of the plastid envelope: an update. Front Plant Sci. 2011:2:50. 10.3389/fpls.2011.00050. 22645538 PMC3355759

[msae012-B33] Fan X, Qiu H, Han W, Wang Y, Xu D, Zhang X, Bhattacharya D, Ye N. Phytoplankton pangenome reveals extensive prokaryotic horizontal gene transfer of diverse functions. Sci Adv. 2020:6(18):eaba0111. 10.1126/sciadv.aba0111. 32494685 PMC7190310

[msae012-B34] Fang G, Yu H, Kirschner MW. Direct binding of CDC20 protein family members activates the anaphase-promoting complex in mitosis and G1. Mol Cell. 1998:2(2):163–171. 10.1016/S1097-2765(00)80126-4. 9734353

[msae012-B35] Farinas B, Mas P. Functional implication of the MYB transcription factor RVE8/LCL5 in the circadian control of histone acetylation. Plant J. 2011:66(2):318–329. 10.1111/j.1365-313X.2011.04484.x. 21205033

[msae012-B36] Fogelmark K, Troein C. Rethinking transcriptional activation in the *Arabidopsis* circadian clock. PLoS Comput Biol. 2014:10(7):e1003705. 10.1371/journal.pcbi.1003705. 25033214 PMC4102396

[msae012-B37] Fortunato AE, Annunziata R, Jaubert M, Bouly JP, Falciatore A. Dealing with light: the widespread and multitasking cryptochrome/photolyase family in photosynthetic organisms. J Plant Physiol. 2015:172:42–54. 10.1016/j.jplph.2014.06.011. 25087009

[msae012-B38] Froehlich AC, Chen C-H, Belden W, Madeti C, Roenneberg T, Merrow M, Loros JJ, Dunlap JC. Genetic and molecular characterization of a cryptochrome from the filamentous fungus *Neurospora crassa*. Eukaryot Cell. 2010:9(5):738–750. 10.1128/EC.00380-09. 20305004 PMC2863965

[msae012-B39] Gray JA, Shalit-Kaneh A, Chu DN, Hsu PY, Harmer SL. The REVEILLE clock genes inhibit growth of juvenile and adult plants by control of cell size. Plant Physiol. 2017:173(4):2308–2322. 10.1104/pp.17.00109. 28254761 PMC5373068

[msae012-B40] Gurrieri L, Fermani S, Zaffagnini M, Sparla F, Trost P. Calvin-Benson cycle regulation is getting complex. Trends Plant Sci. 2021:26(9):898–912. 10.1016/j.tplants.2021.03.008. 33893047

[msae012-B41] Han X, Chang X, Zhang Z, Chen H, He H, Zhong B, Deng XW. Origin and evolution of core components responsible for monitoring light environment changes during plant terrestrialization. Mol Plant. 2019:12(6):847–862. 10.1016/j.molp.2019.04.006. 31009752

[msae012-B42] Harmer SL . The circadian system in higher plants. Annu Rev Plant Biol. 2009:60:357–377. 10.1146/annurev.arplant.043008.092054. 19575587

[msae012-B43] Hatch MD, Kagawa T. Activity, location and role of NAD malic enzyme in leaves with C_4_-pathway photosynthesis. Funct Plant Biol. 1974:1(3):357–369. 10.1071/PP9740357.

[msae012-B44] Holtkotte X, Ponnu J, Ahmad M, Hoecker U. The blue light-induced interaction of cryptochrome 1 with COP1 requires SPA proteins during *Arabidopsis* light signaling. PLoS Genet. 2017:13(10):e1007044. 10.1371/journal.pgen.1007044. 28991901 PMC5648270

[msae012-B45] Huang J, Mullapudi NN, Lancto CA, Scott M, Abrahamsen MS, Kissinger JC. Phylogenomic evidence supports past endosymbiosis, intracellular and horizontal gene transfer in *Crytosporidium parvum*. Genome Biol. 2004:5(11):R88. 10.1186/gb-2004-5-11-r88. 15535864 PMC545779

[msae012-B46] Huerta-Cepas J, Forslund K, Coelho LP, Szklarczyk D, Jensen LJ, von Mering C, Pork P. Fast genome-wide functional annotation through orthology assignment by eggNOG-mapper. Mol Biol Evol. 2017:34(8):2115–2122. 10.1093/molbev/msx148. 28460117 PMC5850834

[msae012-B47] Hughes RM, Vrana JD, Song J, Tucker CL. Light-dependent, dark-promoted interaction between *Arabidopsis* cryptochrome 1 and phytochrome B proteins. J Biol Chem. 2012:287(26):22165–22172. 10.1074/jbc.M112.360545. 22577138 PMC3381176

[msae012-B48] Husnik F, McCutcheon JP. Functional horizontal gene transfer from bacteria to eukaryotes. Nat Rev Microbiol. 2017:16(2):67–79. 10.1038/nrmicro.2017.137. 29176581

[msae012-B49] Ichikawa K, Sugita M, Imaizumi T, Wada M, Aoki S. Differential expression on a daily basis of plastid sigma factor genes from the moss *Physcomitrella patens*. Regulatory interactions among PpSig5, the circadian clock, and blue light signaling mediated by cryptochromes. Plant Physiol. 2004:136(4):4285–4298. 10.1104/pp.104.053033. 15563615 PMC535858

[msae012-B50] Imaizumi T, Schultz TF, Harmon FG, Ho LA, Kay SA. FKF1 F-box protein mediates cyclic degradation of a repressor of CONSTANS in *Arabidopsis*. Science. 2005:309(5732):293–297. 10.1126/science.1110586. 16002617

[msae012-B51] Johnson LK, Alexander H, Brown CT. Re-assembly, quality evaluation, and annotation of 678 microbial eukaryotic reference transcriptomes. GigaScience. 2019:8(4):giy158. 10.1093/gigascience/giy158. 30544207 PMC6481552

[msae012-B52] Jones CR, Huang AL, Ptáček LJ, Fu Y-H. Genetic basis of human circadian rhythm disorders. Exp Neurol. 2013:243:28–33. 10.1016/j.expneurol.2012.07.012. 22849821 PMC3514403

[msae012-B53] Juhas M, von Zadow A, Spexard M, Schmidt M, Kottke T, Büchel C. A novel cryptochrome in the diatom *Phaeodactylum tricornutum* influences the regulation of light-harvesting protein levels. FEBS J. 2014:281(9):2299–2311. 10.1111/febs.12782. 24628952

[msae012-B54] Keeling PJ, Burki F, Wilcox HM, Allam B, Allen EE, Amaral-Zettler LA, Armbrust EV, Archibald JM, Bharti AK, Bell CJ, et al The Marine Microbial Eukaryote Transcriptome Sequencing Project (MMETSP): illuminating the functional diversity of eukaryotic life in the oceans through transcriptome sequencing. PLoS Biol. 2014:12(6):e1001889. 10.1371/journal.pbio.1001889. 24959919 PMC4068987

[msae012-B55] Khoshravesh R, Stata M, Adachi S, Sage TL, Sage RF. Evolutionary convergence of C3 photosynthesis: a case study in the Nyctaginaceae. Front Plant Sci. 2020:11:578739. 10.3389/fpls.2020.578739. 33224166 PMC7667235

[msae012-B56] Kianianmomeni A, Hallmann A. Algal photoreceptors: in vivo functions and potential applications. Planta. 2014:239(1):1–26. 10.1007/s00425-013-1962-5. 24081482

[msae012-B57] Kim D, Pertea G, Trapnell C, Pimentel H, Kelley R, Salzberg SL. TopHat2: accurate alignment of transcriptomes in the presence of insertions, deletions and gene fusions. Genome Biol. 2013:14(4):R36. 10.1186/gb-2013-14-4-r36. 23618408 PMC4053844

[msae012-B58] Kim JY, Song JT, Seo HS. COP1 regulates plant growth and development in response to light at the post-translational level. J Exp Bot. 2017:68(17):4737–4748. 10.1093/jxb/erx312. 28992300

[msae012-B59] Kiontke S, Göbel T, Brych A, Batschauer A. DASH-type cryptochromes—solved and open questions. Biol Chem. 2020:401(12):1487–1493. 10.1515/hsz-2020-0182. 32663167

[msae012-B60] Kleine T, Lockhart P, Batschauer A. An *Arabidopsis* protein closely related to *Synechocystis* cryptochrome is targeted to organelles. Plant J. 2003:35(1):93–103. 10.1046/j.1365-313X.2003.01787.x. 12834405

[msae012-B61] Kondo T, Ishiura M. The circadian clock of cyanobacteria. BioEssays. 2000:22(1):10–15. 10.1002/(SICI)1521-1878(200001)22:1<10::AID-BIES4>3.0.CO;2-A. 10649285

[msae012-B62] Krause-Jensen D, Lavery P, Serrano O, Marbà N, Masque P, Duarte CM. Sequestration of macroalgal carbon: the elephant in the blue carbon room. Biol Lett. 2018:14(6):20180236. 10.1098/rsbl.2018.0236. 29925564 PMC6030603

[msae012-B63] Krylov VV, Izvekov EI, Pavlova VV, Pankova NA, Osipova EA. Circadian rhythms in zebrafish (*Danio rerio*) behavior and the sources of their variability. Biol Rev. 2021:96(3):785–797. 10.1111/brv.12678. 33331134

[msae012-B64] Kühlbrandt W . Structure and mechanisms of F-type ATP synthases. Annu Rev Biochem. 2019:88(1):515–549. 10.1146/annurev-biochem-013118-110903. 30901262

[msae012-B65] Lai LB, Tausta SL, Nelson TM. Differential regulation of transcripts encoding cytosolic NADP-malic enzyme in C_3_ and C_3_ *Flaveria* species. Plant Physiol. 2002:128(1):140–149. 10.1104/pp.010449. 11788759 PMC148956

[msae012-B66] Lee JM . Algal genomics perspective: the pangenome concept beyond traditional molecular phylogeny and taxonomy. J Species Res. 2021:10(2):142–153. 10.12651/JSR.2021.10.2.142.

[msae012-B67] Lee JM, Cho CH, Park SI, Choi JW, Song HS, West JA, Bhattacharya D, Yoon HS. Parallel evolution of highly conserved plastid genome architecture in red seaweeds and seed plants. BMC Biol. 2016:14(1):75. 10.1186/s12915-016-0299-5. 27589960 PMC5010701

[msae012-B68] Lee S, Uchida Y, Wang J, Matsudaira T, Nakagawa T, Kishimoto T, Mukai K, Inaba T, Kobayashi T, Molday RS, et al Transport through recycling endosomes requires EHD1 recruitment by a phosphatidylserine translocase. EMBO J. 2015:34(5):669–688. 10.15252/embj.201489703. 25595798 PMC4365035

[msae012-B69] Lee JM, Yang EC, Graf L, Yang JH, Qiu H, Zelzion U, Chan CX, Stephens TG, Weber APM, Boo GH, et al Analysis of the draft genome of the red seaweed *Gracilariopsis chorda* provides insights into genome size evolution in Rhodophyta. Mol Biol Evol. 2018:35(8):1869–1886. 10.1093/molbev/msy081. 29688518

[msae012-B70] Leegood RC, Walker RP. Regulation and roles of phosphoenolpyruvate carboxykinase in plants. Arch Biochem Biophys. 2003:414(2):204–210. 10.1016/S0003-9861(03)00093-6. 12781772

[msae012-B71] Lhee D, Lee JM, Ettahi K, Cho CH, Ha J-S, Chan Y-F, Zelzion U, Stephens TG, Price DC, Gabr A, et al Amoeba genome reveals dominant host contribution to plastid endosymbiosis. Mol Biol Evol. 2020:38(2):344–357. 10.1093/molbev/msaa206. PMC7826189 32790833

[msae012-B72] Li P, Liao Z, Zhou J, Yin L, Jiang HS, Li W. Bicarbonate-use by aquatic macrophytes allows a reduction in photorespiration at low CO_2_ concentrations. Environ Exp Bot. 2021:188:104520. 10.1016/j.envexpbot.2021.104520.

[msae012-B73] Linde A-M, Eklund DM, Kubota A, Pederson ERA, Holm K, Gyllenstrand N, Nishihama R, Cronberg N, Muranaka T, Oyama T, et al Early evolution of the land plant circadian clock. New Phytol. 2017:216(2):576–590. 10.1111/nph.14487. 28244104 PMC5638080

[msae012-B74] Lindskog S . Structure and mechanism of carbonic anhydrase. Pharmacol Ther. 1997:74(1):1–20. 10.1016/S0163-7258(96)00198-2. 9336012

[msae012-B75] Liu Y, Sun Y, Yao H, Zheng Y, Cao S, Wang H. *Arabidopsis* circadian clock repress phytochrome a signaling. Front Plant Sci. 2022:13:809563. 10.3389/fpls.2022.809563. 35645991 PMC9131076

[msae012-B76] Lopez L, Fasano C, Perrella G, Facella P. Cryptochromes and the circadian clock: the story of a very complex relationship in a spinning world. Genes (Basel). 2021:12(5):672. 10.3390/genes12050672. 33946956 PMC8145066

[msae012-B77] Macreadie PI, Anton A, Raven JA, Beaumont N, Connolly RM, Friess DA, Kelleway JK, Kennedy H, Kuwae T, Lavery PS, et al The future of blue carbon science. Nat Commun. 2019:10(1):3998. 10.1038/s41467-019-11693-w. 31488846 PMC6728345

[msae012-B78] Maier A, Zell MB, Maurino VG. Malate decarboxylases: evolution and roles of NAD(P)-ME isoforms in species performing C4 and C3 photosynthesis. J Exp Bot. 2011:62(9):3061–3069. 10.1093/jxb/err024. 21459769

[msae012-B79] Marchler-Bauer A, Bo Y, Han L, He J, Lanczycki CJ, Lu S, Chitsaz F, Derbyshire MK, Geer RC, Gonzales NR, et al CDD/SPARCLE: functional classification of proteins via subfamily domain architectures. Nucleic Acids Res. 2017:45(D1):D200–D203. 10.1093/nar/gkw1129. 27899674 PMC5210587

[msae012-B80] Matsuo T, Ishiura M. *Chlamydomonas reinhardtii* as a new model system for studying the molecular basis of the circadian clock. FEBS Lett. 2011:585(10):1495–1502. 10.1016/j.febslet.2011.02.025. 21354416

[msae012-B81] Matsuo T, Okamoto K, Onai K, Niwa Y, Shimogawara K, Ishiura M. A systematic forward genetic analysis identified components of the *Chlamydomonas* circadian system. Genes Dev. 2008:22(7):918–930. 10.1101/gad.1650408. 18334618 PMC2279203

[msae012-B82] McClung CR . The plant circadian oscillator. Biology (Basel). 2019:8(1):14. 10.3390/biology8010014. 30870980 PMC6466001

[msae012-B83] Michael AK, Fribourgh JL, Van Gelder RN, Partch CL. Animal cryptochromes: divergent roles in light perception, circadian timekeeping and beyond. Photochem Photobiol. 2017:93(1):128–140. 10.1111/php.12677. 27891621 PMC5397253

[msae012-B84] Miyagishima S, Fujiwara T, Sumiya N, Hirooka S, Nakano A, Kabeya Y, Nakamura M. Translation-independent circadian control of the cell cycle in a unicellular photosynthetic eukaryote. Nat Commun. 2014:5(1):3807. 10.1038/ncomms4807. 24806410

[msae012-B85] Nakamichi N . The transcriptional network in the *Arabidopsis* circadian clock system. Genes (Basel). 2020:11(11):1284. 10.3390/genes11111284. 33138078 PMC7692566

[msae012-B86] Nguyen LT, Schmidt HA, von Haeseler A, Minh BQ. IQ-TREE: a fast and effective stochastic algorithm for estimating maximum-likelihood phylogenies. Mol Biol Evol. 2015:32(1):268–274. 10.1093/molbev/msu300. 25371430 PMC4271533

[msae012-B87] Nielsen H, Engelbrecht J, Brunak S, von Heijne G. Identification of prokaryotic and eukaryotic signal peptides and prediction of their cleavage sites. Protein Eng. 1997:10(1):1–6. 10.1093/protein/10.1.1. 9051728

[msae012-B88] Nitabach MN, Taghert PH. Organization of the *Drosophila* circadian control circuit. Curr Biol. 2008:18(2):R84–R93. 10.1016/j.cub.2007.11.061. 18211849

[msae012-B89] Noordally ZB, Ishii K, Atkins KA, Wetherill SJ, Kusakina J, Walton EJ, Kato M, Azuma M, Tanaka K, Hanaoka M, et al Circadian control of chloroplast transcription by a nuclear-encoded timing signal. Science. 2013:339(6125):1316–1319. 10.1126/science.1230397. 23493713

[msae012-B90] Noordally ZB, Millar AJ. Clocks in algae. Biochemistry. 2014:54(2):171–183. 10.1021/bi501089x. 25379817

[msae012-B91] Nowack ECM, Melkonian M, Glöckner G. Chromatophore genome sequence of *Paulinella* sheds light on acquisition of photosynthesis by eukaryotes. Curr Biol. 2008:18(6):410–418. 10.1016/j.cub.2008.02.051. 18356055

[msae012-B92] Ohgishi M, Saji K, Okada K, Sakai T. Functional analysis of each blue light receptor, cry1, cry2, phot1, and phot2, by using combinatorial multiple mutants in *Arabidopsis*. Proc Natl Acad Sci U S A. 2004:101(8):2223–2228. 10.1073/pnas.0305984101. 14982991 PMC356932

[msae012-B93] Patro R, Duggal G, Love MI, Irizarry RA, Kingsford C. Salmon provides fast and bias-aware quantification of transcript expression. Nat Methods. 2017:14(4):417–419. 10.1038/nmeth.4197. 28263959 PMC5600148

[msae012-B94] Patron NJ, Keeling PJ. Common evolutionary origin of starch biosynthetic enzymes in green and red algae. J Phycol. 2005:41(6):1131–1141. 10.1111/j.1529-8817.2005.00135.x.

[msae012-B95] Perdomo JA, Buchner P, Carmo-Silva E. The relative abundance of wheat Rubisco activase isoforms is post-transcriptionally regulated. Photosynth Res. 2021:148(1-2):47–56. 10.1007/s11120-021-00830-6. 33796933 PMC8154801

[msae012-B96] Pereira L, Christin P-A, Dunning LT. The mechanisms underpinning lateral gene transfer between grasses. Plants People Planet. 2022:5(5):672–682. 10.1002/ppp3.10347.

[msae012-B97] Petersen J, Rredhi A, Szyttenholm J, Oldemeyer S, Kottke T, Mittag M. The world of algae reveals a broad variety of cryptochrome properties and functions. Front Plant Sci. 2021:12:766509. 10.3389/fpls.2021.766509. 34790217 PMC8591175

[msae012-B98] Piechura JR, Amarnath K, O'Shea EK. Natural changes in light interact with circadian regulation at promoters to control gene expression in cyanobacteria. eLife. 2017:6:e32032. 10.7554/eLife.32032. 29239721 PMC5785211

[msae012-B99] Ponnu J, Hoecker U. Illuminating the COP1/SPA ubiquitin ligase: fresh insights into its structure and functions during plant photomorphogenesis. Front Plant Sci. 2021:12:662793. 10.3389/fpls.2021.662793. 33841486 PMC8024647

[msae012-B100] Poschenrieder C, Fernández JA, Rubio L, Pérez L, Terés J, Barceló J. Transport and use of bicarbonate in plants: current knowledge and challenges ahead. Int J Mol Sci. 2018:19(5):1352. 10.3390/ijms19051352. 29751549 PMC5983714

[msae012-B101] Raghavendra AS, Padmasree K. Beneficial interactions of mitochondrial metabolism with photosynthetic carbon assimilation. Trends Plant Sci. 2003:8(11):546–553. 10.1016/j.tplants.2003.09.015. 14607100

[msae012-B102] Rao X, Dixon RA. The differences between NAD-ME and NADP-ME subtypes of C4 photosynthesis: more than decarboxylating enzymes. Front Plant Sci. 2016:7:1525. 10.3389/fpls.2016.01525. 27790235 PMC5061750

[msae012-B103] Raven JA . Exogenous inorganic carbon sources in plant photosynthesis. Biol Rev. 1970:45(2):167–220. 10.1111/j.1469-185X.1970.tb01629.x.

[msae012-B104] Raven JA, Beardall J. The ins and outs of CO_2_. J Exp Bot. 2016:67(1):1–13. 10.1093/jxb/erv451. 26466660 PMC4682431

[msae012-B105] Rawat R, Takahashi N, Hsu PY, Jones MA, Schwartz J, Salemi MR, Phinney BS, Harmer SL. REVEILLE8 and pseudo-reponse regulator5 form a negative feedback loop within the *Arabidopsis* circadian clock. PLoS Genet. 2011:7(3):e1001350. 10.1371/journal.pgen.1001350. 21483796 PMC3069099

[msae012-B106] Razzak MA, Lee DW, Lee J, Hwang I. Overexpression and purification of *Gracilariopsis chorda* carbonic anhydrase (GcCAα3) in *Nicotiana benthamiana*, and its immobilization and use in CO_2_ hydration reactions. Front Plant Sci. 2020:11:563721. 10.3389/fpls.2020.563721. 33329625 PMC7717956

[msae012-B107] Razzak MA, Lee JM, Lee DW, Kim JH, Yoon HS, Hwang I. Expression of seven carbonic anhydrases in red alga *Gracilariopsis chorda* and their subcellular localization in a heterologous system, *Arabidopsis thaliana*. Plant Cell Rep. 2018:38(2):147–159. 10.1007/s00299-018-2356-8. 30446790

[msae012-B108] Recuenco-Muñoz L, Offre P, Valledor L, Lyon D, Weckwerth W, Wienkoop S. Targeted quantitative analysis of a diurnal RuBisCO subunit expression and translation profile in *Chlamydomonas reinhardtii* introducing a novel Mass Western approach. J Proteomics. 2015:113:143–153. 10.1016/j.jprot.2014.09.026. 25301535

[msae012-B109] Rockwell NC, Lagarias JC. Phytochrome diversification in cyanobacteria and eukaryotic algae. Curr Opin Plant Biol. 2017:37:87–93. 10.1016/j.pbi.2017.04.003. 28445833 PMC5483197

[msae012-B110] Rockwell NC, Lagarias JC. Phytochrome evolution in 3D: deletion, duplication, and diversification. New Phytol. 2020:225(6):2283–2300. 10.1111/nph.16240. 31595505 PMC7028483

[msae012-B111] Rossoni AW, Price DC, Seger M, Lyska D, Lammers P, Bhattacharya D, Weber APM. The genomes of polyextremophilic Cyanidiales contain 1% horizontally transferred genes with diverse adaptive functions. eLife. 2019:8:e45017. 10.7554/eLife.45017. 31149898 PMC6629376

[msae012-B112] Rredhi A, Petersen J, Schubert M, Li W, Oldemeyer S, Li W, Westermann M, Wagner V, Kottke T, Mittag M. DASH cryptochrome 1, a UV-A receptor, balances the photosynthetic machinery of *Chlamydomonas reinhardtii*. New Phytol. 2021:232(2):610–624. 10.1111/nph.17603. 34235760

[msae012-B113] Sage RF, Sage TL, Kocacinar F. Photorespiration and the evolution of C_4_ photosynthesis. Annu Rev Plant Biol. 2012:63(1):19–47. 10.1146/annurev-arplant-042811-105511. 22404472

[msae012-B114] Sawa M, Nusinow DA, Kay SA, Imaizumi T. FKF1 and GIGANTEA complex formation is required for day-length measurement in *Arabidopsis*. Science. 2007:318(5848):261–265. 10.1126/science.1146994. 17872410 PMC3709017

[msae012-B115] Schaffer R, Ramsay N, Samach A, Corden S, Putterill J, Carré IA, Coupland G. The *late elongated hypocotyl* mutation of *Arabidopsis* disrupts circadian rhythms and the photoperiodic control of flowering. Cell. 1998:93(7):1219–1229. 10.1016/S0092-8674(00)81465-8. 9657154

[msae012-B116] Schönknecht G, Chen W, Ternes CM, Barbier GG, Shrestha RP, Stanke M, Bräutigam A, Baker BJ, Banfield JF, Garavito RM, et al Gene transfer from Bacteria and Archaea facilitated evolution of an extremophilic eukaryote. Science. 2013:339(6124):1207–1210. 10.1126/science.1231707. 23471408

[msae012-B117] Serrano-Bueno G, Romero-Campero FJ, Lucas-Reina E, Romero JM, Valverde F. Evolution of photoperiod sensing in plants and algae. Curr Opin Plant Biol. 2017:37:10–17. 10.1016/j.pbi.2017.03.007. 28391047

[msae012-B118] Shen Z, Dong X-M, Gao Z-F, Chao Q, Wang B-C. Phylogenic and phosphorylation regulation difference of phosphoenolypyruvate carboxykinase of C3 and C4 plants. J Plant Physiol. 2017:213:16–22. 10.1016/j.jplph.2017.02.008. 28285130

[msae012-B119] Shi J, Yi K, Liu Y, Xie L, Zhou Z, Chen Y, Hu Z, Zheng T, Liu R, Chen Y, et al Phosphoenolpyruvate carboxylase in *Arabidopsis* leaves plays a crucial role in carbon and nitrogen metabolism. Plant Physiol. 2015:167(3):671–681. 10.1104/pp.114.254474. 25588735 PMC4348777

[msae012-B120] Shu J-P, Yan Y-H, Wang R-J. Convergent molecular evolution of phosphoenolpyruvate carboxylase gene family in C_4_ and crassulacean acid metabolism plants. PeerJ. 2022:10:e12828. 10.7717/peerj.12828. 35116203 PMC8784020

[msae012-B121] Smith DR, Lima MS. Unraveling chloroplast transcriptomes with ChloroSeq, an organelle RNA-Seq bioinformatics pipeline. Brief Bioinform. 2017:18(6):1012–1016. 10.1093/bib/bbw088. 27677960 PMC5862312

[msae012-B122] Taddei L, Stella GR, Rogato A, Bailleul B, Fortunato AE, Annunziata R, Sanges R, Thaler M, Lepetit B, Lavaud J, et al Multisignal control of expression of the LHCX protein family in the marine diatom *Phaeodactylum tricornutum*. J Exp Bot. 2016:67(13):3939–3951. 10.1093/jxb/erw198. 27225826 PMC4915529

[msae012-B123] Taton A, Erikson C, Yang Y, Rubin BE, Rifkin SA, Golden JW, Golden SS. The circadian clock and darkness control natural competence in cyanobacteria. Nat Commun. 2020:11(1):1688. 10.1038/s41467-020-15384-9. 32245943 PMC7125226

[msae012-B124] Tay IYY, Odang KB, Cheung CYM. Metabolic modeling of the C3-CAM continuum revealed the establishment of a starch/sugar-malate cycle in CAM evolution. Front Plant Sci. 2021:11:573197. 10.3389/fpls.2020.573197. 33584741 PMC7874232

[msae012-B125] Thum KE, Kim M, Christopher DA, Mullet JE. Cryptochrome 1, cryptochrome 2, and phytochrome A co-activate the chloroplast *psb*D blue light-responsive promoter. Plant Cell. 2001:13(12):2747–2760. 10.1105/tpc.010345. 11752385 PMC139486

[msae012-B126] Tronconi MA, Andreo CS, Drincovich MF. Chimeric structure of plant malic enzyme family: different evolutionary scenarios for NAD- and NADP-dependent isoforms. Front Plant Sci. 2018:9:565. 10.3389/fpls.2018.00565. 29868045 PMC5958461

[msae012-B127] Tronconi MA, Fahnenstich H, Weehler MCG, Andreo CS, Flügge UI, Drincovish MF, Maurino VG. *Arabidopsis* NAD-malic enzyme functions as a homodimer and heterodimer and has a major impact on nocturnal metabolism. Plant Physiol. 2008:146(4):1540–1552. 10.1104/pp.107.114975. 18223148 PMC2287332

[msae012-B128] Van Etten J, Bhattacharya D. Horizontal gene transfer in eukaryotes: not if, but how much? Trends Genet. 2020:36(12):915–925. 10.1016/j.tig.2020.08.006. 33012528

[msae012-B129] Vass IZ, Kós PB, Knoppová J, Komenda J, Vass I. The cry-DASH cryptochrome encoded by the *sll*1629 gene in the cyanobacterium *Synechocystis* PCC 6803 is required for photosystem II repair. J Photochem Photobiol B, Biol. 2014:130:318–326. 10.1016/j.jphotobiol.2013.12.006. 24389045

[msae012-B130] Viola R, Nyvall P, Pedersén M. The unique features of starch metabolism in red algae. Proc R Soc Lond B Biol Sci. 2001:268(1474):1417–1422. 10.1098/rspb.2001.1644. PMC1088757 11429143

[msae012-B131] Walker RP, Chen Z-H, Famiani F. Gluconeogenesis in plants: a key interface between organic acid/amino acid/lipid and sugar metabolism. Molecules. 2021:26(17):5129. 10.3390/molecules26175129. 34500562 PMC8434439

[msae012-B132] Wang Y, Bräutigam A, Weber APM, Zhu X-G. Three distinct biochemical subtypes of C4 photosynthesis? A modelling analysis. J Exp Bot. 2014:65(13):3567–3578. 10.1093/jxb/eru058. 24609651 PMC4085956

[msae012-B133] Wang Q, Lin C. Mechanisms of cryptochrome-mediated photoresponses in plants. Annu Rev Plant Biol. 2020:71(1):23.1–23.27. 10.1146/annurev-arplant-050718-100300. PMC7428154 32169020

[msae012-B134] Wang H, Ma L-G, Li J-M, Zhao H-Y, Deng XW. Direct interaction of *Arabidopsis* cryptochromes with COP1 in light control development. Science. 2001:294(5540):154–158. 10.1126/science.1063630. 11509693

[msae012-B135] Wang Z-Y, Tobin EM. Constitutive expression of the CIRCADIAN CLOCK ASSOCIATED 1 (CCA1) gene disrupts circadian rhythms and suppresses its own expression. Cell. 1998:93(7):1207–1217. 10.1016/S0092-8674(00)81464-6. 9657153

[msae012-B136] Wheeler MCG, Tronconi MA, Drincovich MF, Andreo CS, Flügge U-I, Maurino VG. A comprehensive analysis of the NADP-malic enzyme gene family of *Arabidopsis*. Plant Physiol. 2005:139(1):39–51. 10.1104/pp.105.065953. 16113210 PMC1203356

[msae012-B137] Whitmore D, Soulkes NS, Strähle U, Sassone-Corsi P. Zebrafish clock rhythmic expression reveals independent peripheral circadian oscillators. Nat Neurosci. 1998:1(8):701–707. 10.1038/3703. 10196586

[msae012-B138] Winter K, Smith JAC. CAM photosynthesis: the acid test. New Phytol. 2022:233(2):559–609. 10.1111/nph.17790. PMC9298356 34637529

[msae012-B139] Wu G, Anafi RC, Hughes ME, Kornacker K, Hogenesch JB. MetaCycle: an integrated R package to evaluate periodicity in large scale data. Bioinformatics. 2016:32(21):3351–3353. 10.1093/bioinformatics/btw405. 27378304 PMC5079475

[msae012-B140] Wu G, Spalding EP. Separate functions for nuclear and cytoplasmic cryptochrome 1 during photomorphogenesis of *Arabidopsis* seedlings. Proc Natl Acad Sci U S A. 2007:104(47):18813–18818. 10.1073/pnas.0705082104. 18003924 PMC2141859

[msae012-B141] Wybouw N, Dermauw W, Tirry L, Stevens C, Grbić M, Feyereisen R, van Leeuwen T. A gene horizontally transferred from bacteria protects arthropods from host plant cyanide poisoning. eLife. 2014:3:e02365. 10.7554/eLife.02365. 24843024 PMC4011162

[msae012-B142] Xu D, Jiang Y, Li F, Holm M, Deng XW. BBX21, an *Arabidopsis* B-box protein, directly activates HY5 and is targeted by COP1 for 26S proteasome-mediated degradation. Proc Natl Acad Sci U S A. 2016:113(27):7655–7660. 10.1073/pnas.1607687113. 27325768 PMC4941485

[msae012-B143] Yamada KD, Tomii K, Katoh K. Application of the MAFFT sequence alignment program to large data-reexamination of the usefulness of chained guide trees. Bioinformatics. 2016:32(21):3246–3251. 10.1093/bioinformatics/btw412. 27378296 PMC5079479

[msae012-B144] Yang X, Li L, Wang X, Yao J, Duan D. Non-coding RNAs participate in the regulation of CRY-DASH in the growth and early development of *Saccharina japonica* (Laminariales, Phaeophyceae). Int J Mol Sci. 2020:21(1):309. 10.3390/ijms21010309. 31906436 PMC6981881

[msae012-B145] Yoo S-D, Cho Y-H, Sheen J. *Arabidopsis* mesophyll protoplasts: a versatile cell system for transient gene expression analysis. Nat Protoc. 2007:2(7):1565–1572. 10.1038/nprot.2007.199. 17585298

[msae012-B146] Yoo S-H, Mohawk JA, Siepka SM, Shan Y, Huh SK, Hong H-K, Kornblum I, Kumar V, Koike N, Xu M, et al Competing E3 ubiqutin ligases determine circadian period by regulated degradation of CRY in nucleus and cytoplasm. Cell. 2013:152(5):1091–1105. 10.1016/j.cell.2013.01.055. 23452855 PMC3694781

[msae012-B147] Yu H . Cdc20: a WD40 activator for a cell cycle degradation machine. Mol Cell. 2007:27(1):3–16. 10.1016/j.molcel.2007.06.009. 17612486

[msae012-B148] Yu X, Sharma B, Gregory BD. The impact of epitranscriptomic marks on post-translational regulation in plants. Brief Funct Genomics. 2021:20(2):113–125. 10.1093/bfgp/elaa021. 33274735

[msae012-B149] Zeebe RE . On the molecular diffusion coefficients of dissolved CO_2_, HCO_3_ ^−^, and CO_3_ ^2−^ and their dependence on isotopic mass. Geochim Cosmochim Acta. 2011:75(9):2483–2498. 10.1016/j.gca.2011.02.010.

[msae012-B150] Zhao Y, Yu H, Zhou JM, Smith SM, Li J. Malate circulation: linking chloroplast metabolism to mitochondrial ROS. Trends Plant Sci. 2020:25(5):446–454. 10.1016/j.tplants.2020.01.010. 32304657

[msae012-B151] Zhou W, Sui Z, Wang J, Hu Y, Kang KH, Hong HR, Niaz Z, Wei H, Du Q, Peng C, et al Effects of sodium bicarbonate concentration on growth, photosynthesis, and carbonic anhydrase activity of macroalgae *Gracilariopsis lemaneiformis*, *Gracilaria vermiculophylla*, and *Gracilaria chouae* (Gracilariales, Rhodophyta). . Photosynth Res. 2016:128(3):259–270. 10.1007/s11120-016-0240-3. 26960545

[msae012-B152] Zones JM, Blaby IK, Merchant SS, Umen JG. High-regulation profiling of a synchronized diurnal transcriptome from *Chlamydomonas reinhardtii* reveals continuous cell and metabolic differentiation. Plant Cell. 2015:27(10):2743–2769. 10.1105/tpc.15.00498. 26432862 PMC4682324

[msae012-B153] Zou Y, Wenzel S, Müller N, Prager K, Jung EM, Kothe E, Kottke T, Mittag M. An animal-like cryptochrome controls the *Chlamydomonas* sexual cycle. Plant Physiol. 2017:174(3):1334–1347. 10.1104/pp.17.00493. 28468769 PMC5490917

