# Supplementary material for: Diurnal Rhythms in the Red Seaweed Gracilariopsis chorda are Characterized by Unique Regulatory Networks of Carbon Metabolism
Source: Mol Biol Evol. 2024 Jan 24;41(2):msae012. doi: 10.1093/molbev/msae012 (PMC10853006; doi:10.1093/molbev/msae012)
Supplement: msae012_Supplementary_Data [file msae012_supplementary_data.zip › Supplementary Figures.pdf]

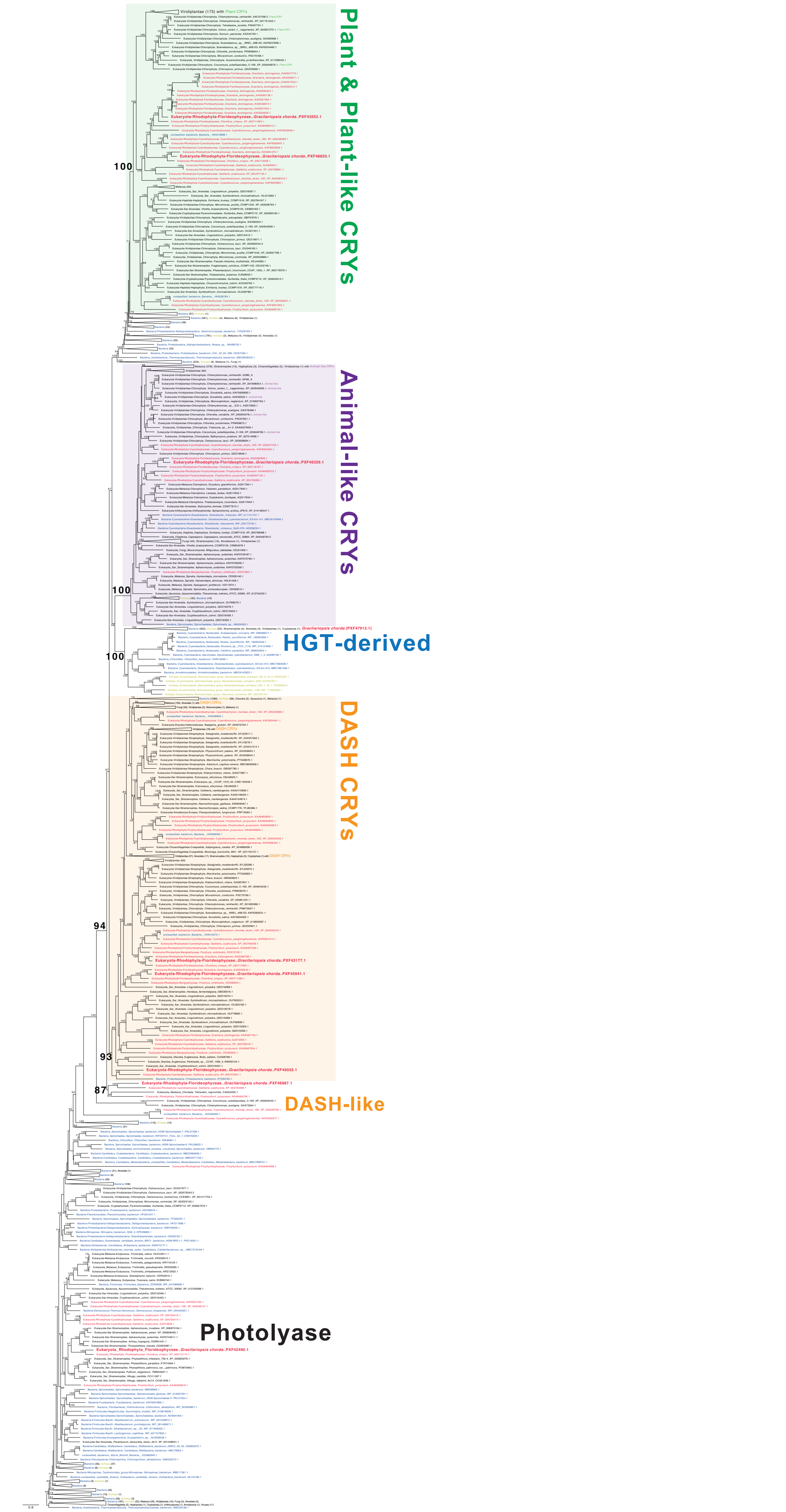

Supplementary Figure S1. The results of a maximum likelihood phylogenetic analysis (using IQ-tree v1.6.12) of cryptochromes. The results of an ultrafast bootstrapping analysis (1,000 replications) are shown on the branches.

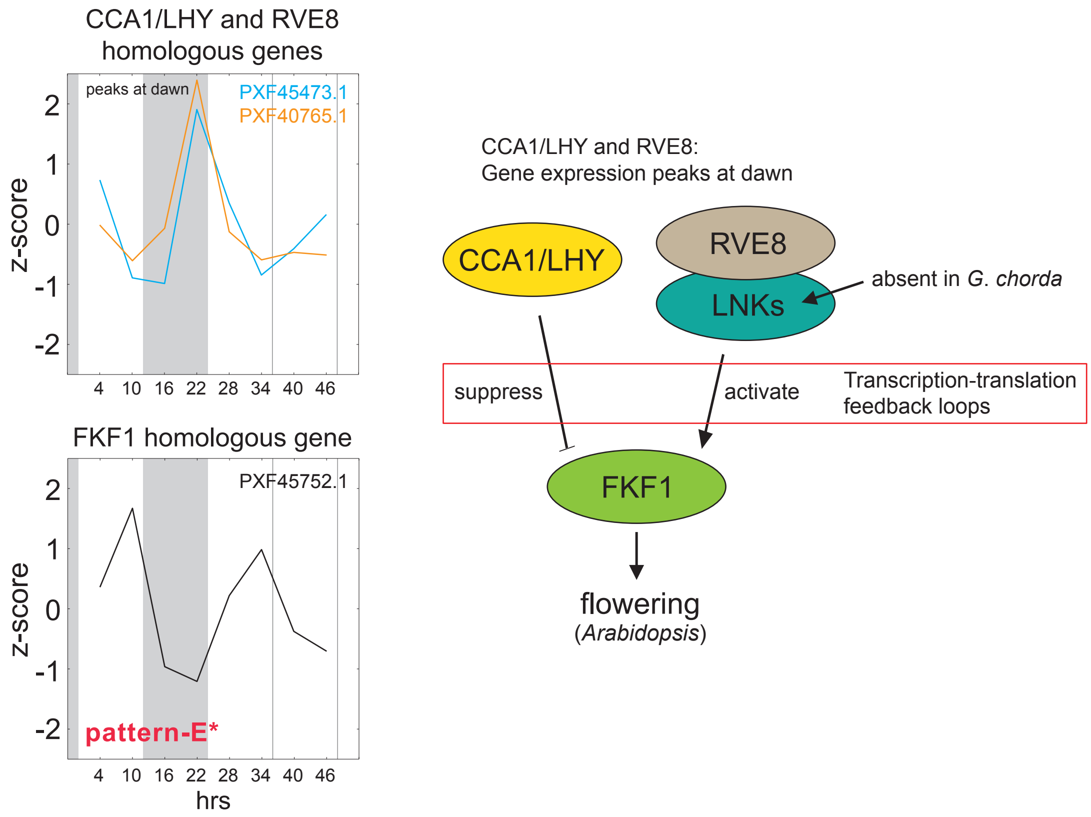

Supplementary Figure S2. Gene expression patterns of CCA1/LHY, RVE8, and FKF1 homologous genes in *G. chorda*, and a simplified model of transcriptional regulation for the suppression and activation of the FKF1 gene in *Arabidopsis*.

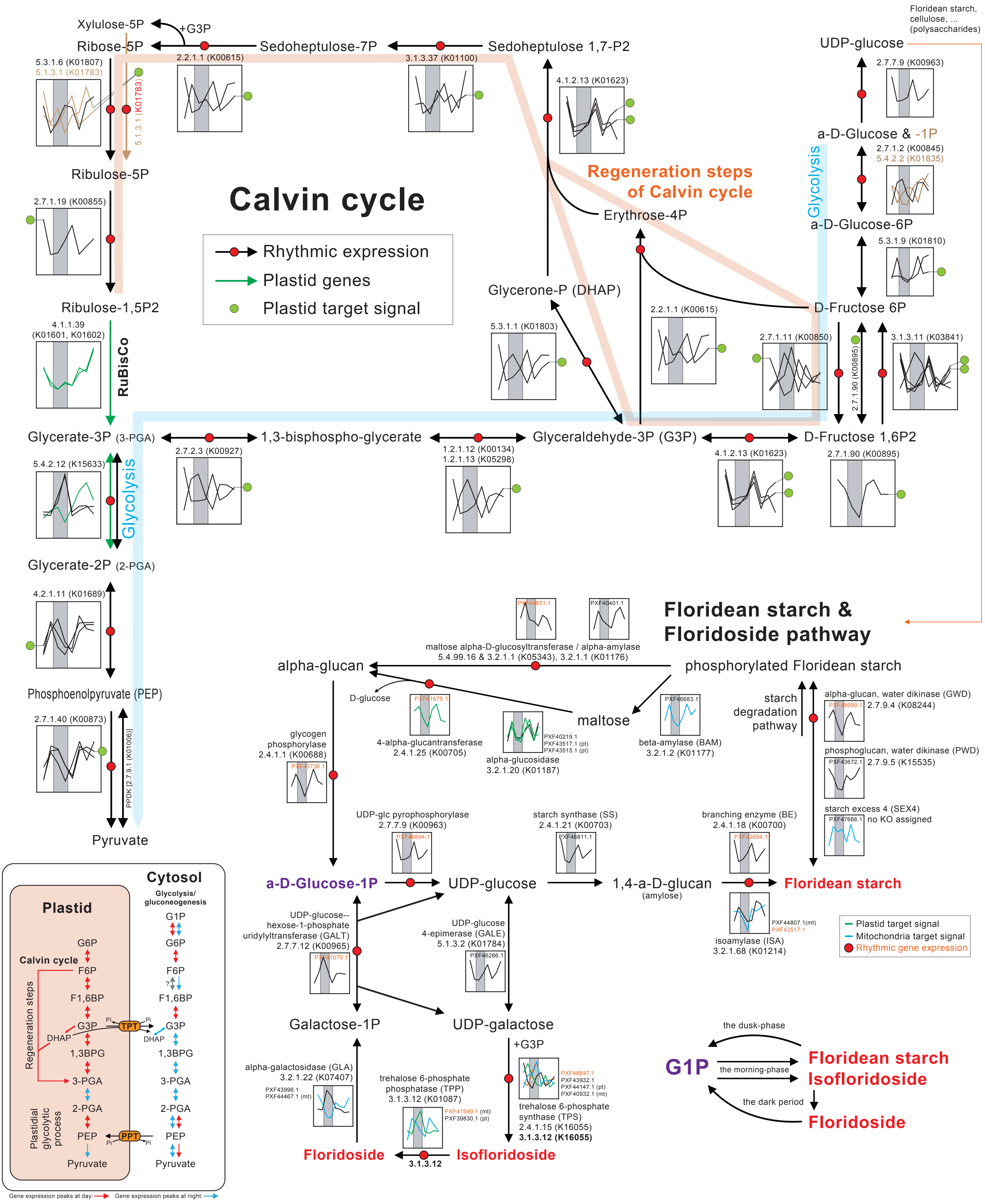

Supplementary Figure S3. Gene expression patterns involving photosynthetic and cytosolic carbon metabolism. Red dots on arrows indicate rhythmic gene expression, and green dots indicate plastid target signal (prediction).

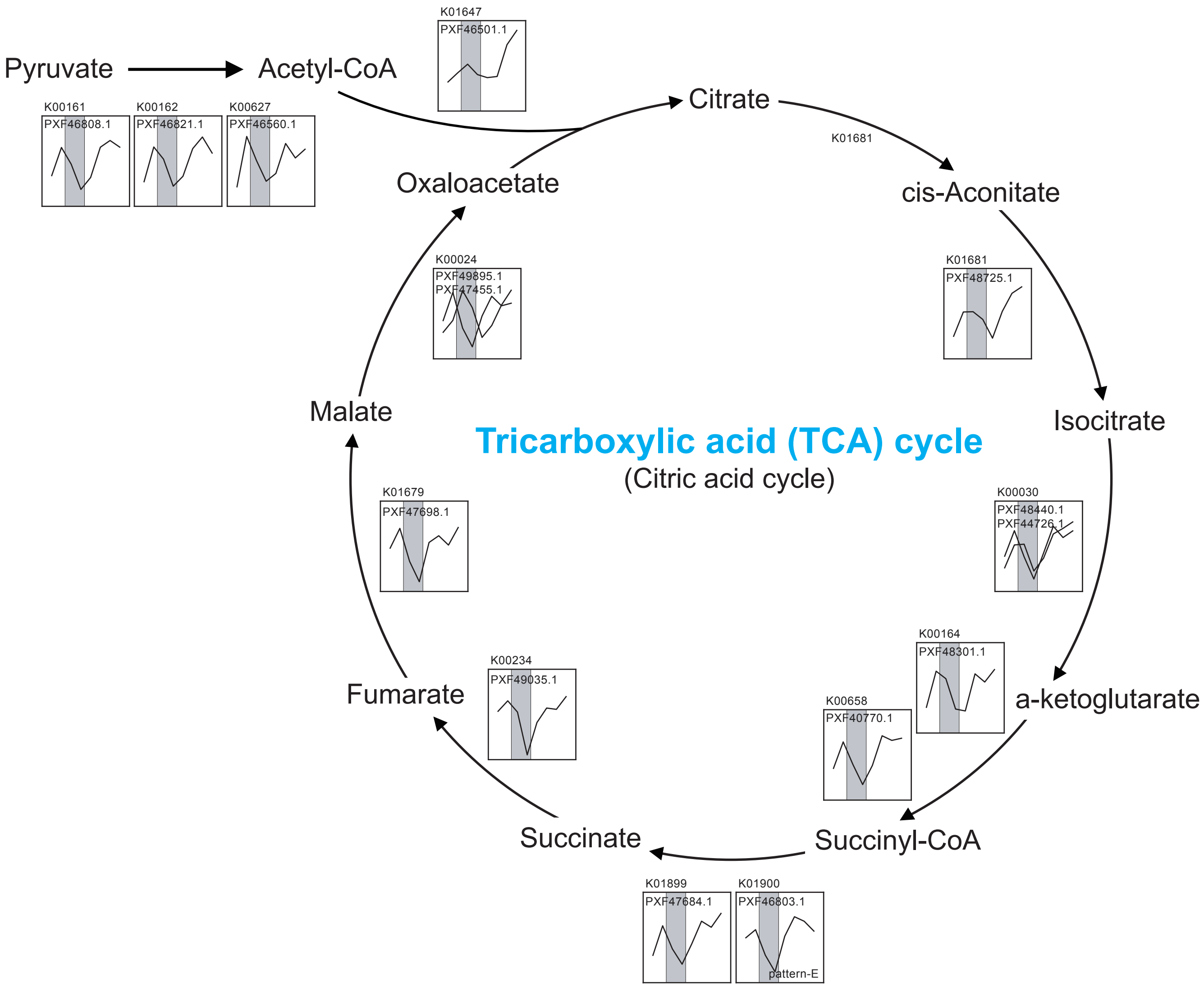

Supplementary Figure S4. Gene expression patterns involving tricarboxylic acid (TCA) cycle in *G. chorda*.

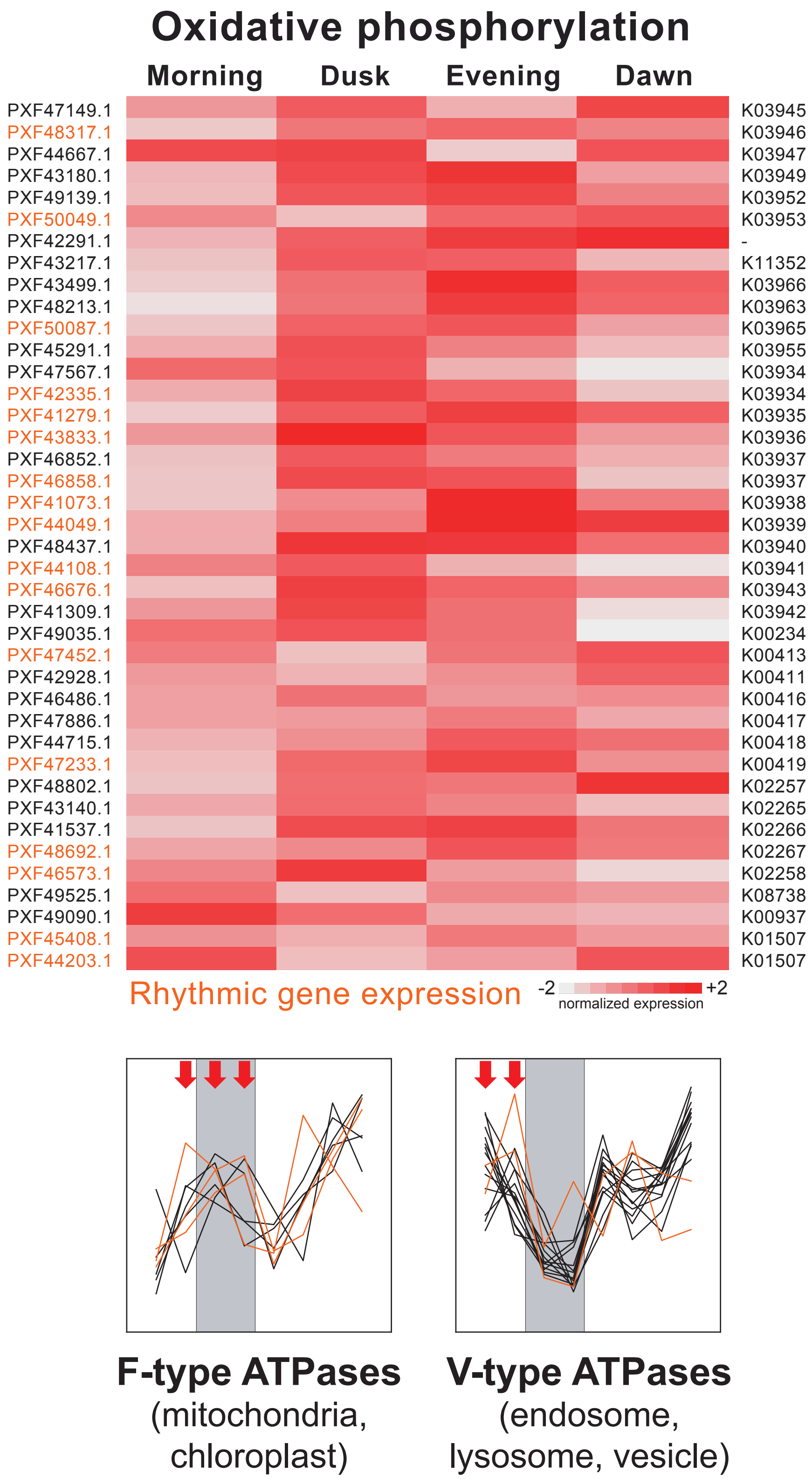

Supplementary Figure S5. Gene expression patterns involving oxidative phosphorylation pathway in *G. chorda*.

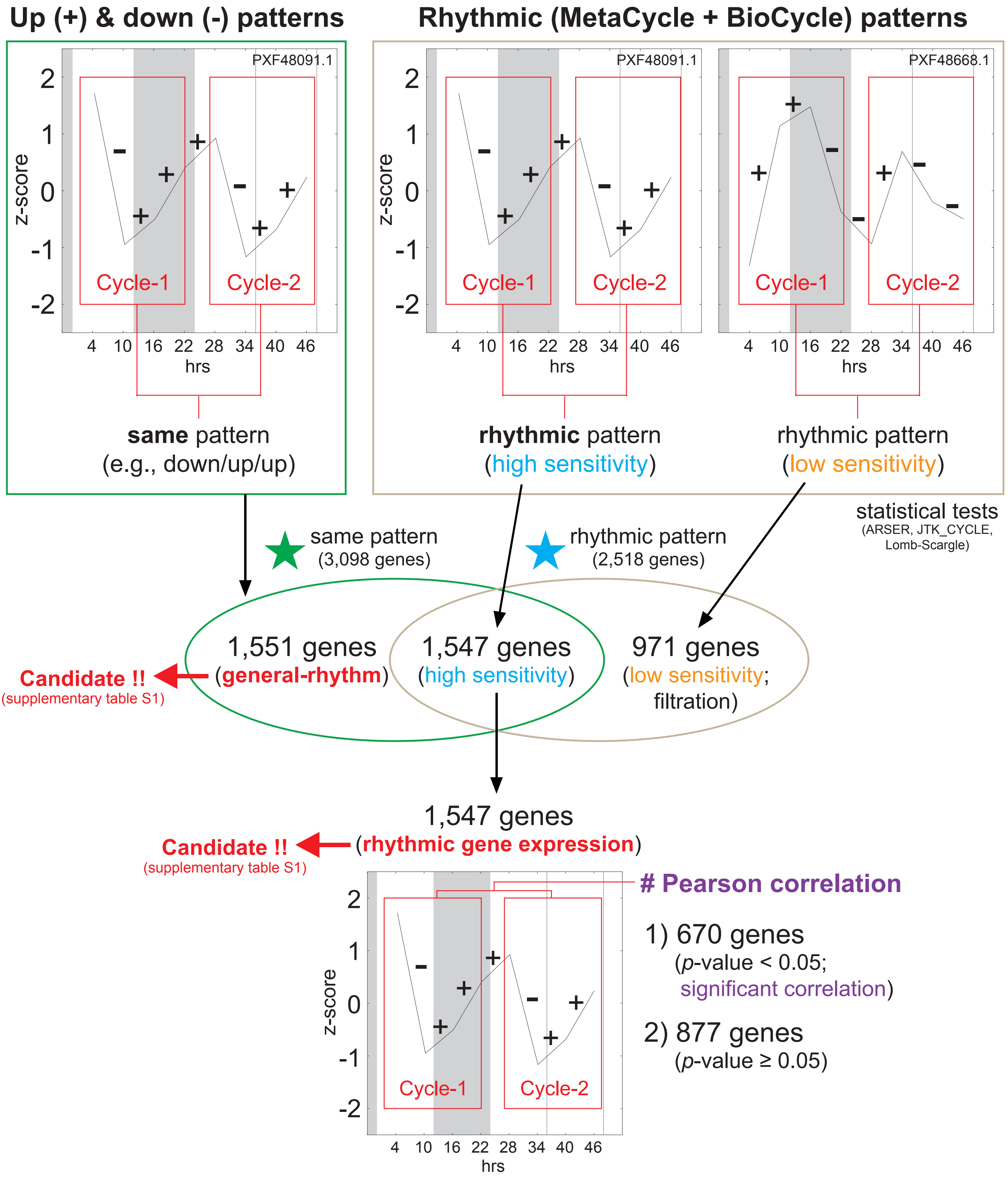

Supplementary Figure S6. Analysis workflow of rhythmic gene expression patterns in *G. chorda*.

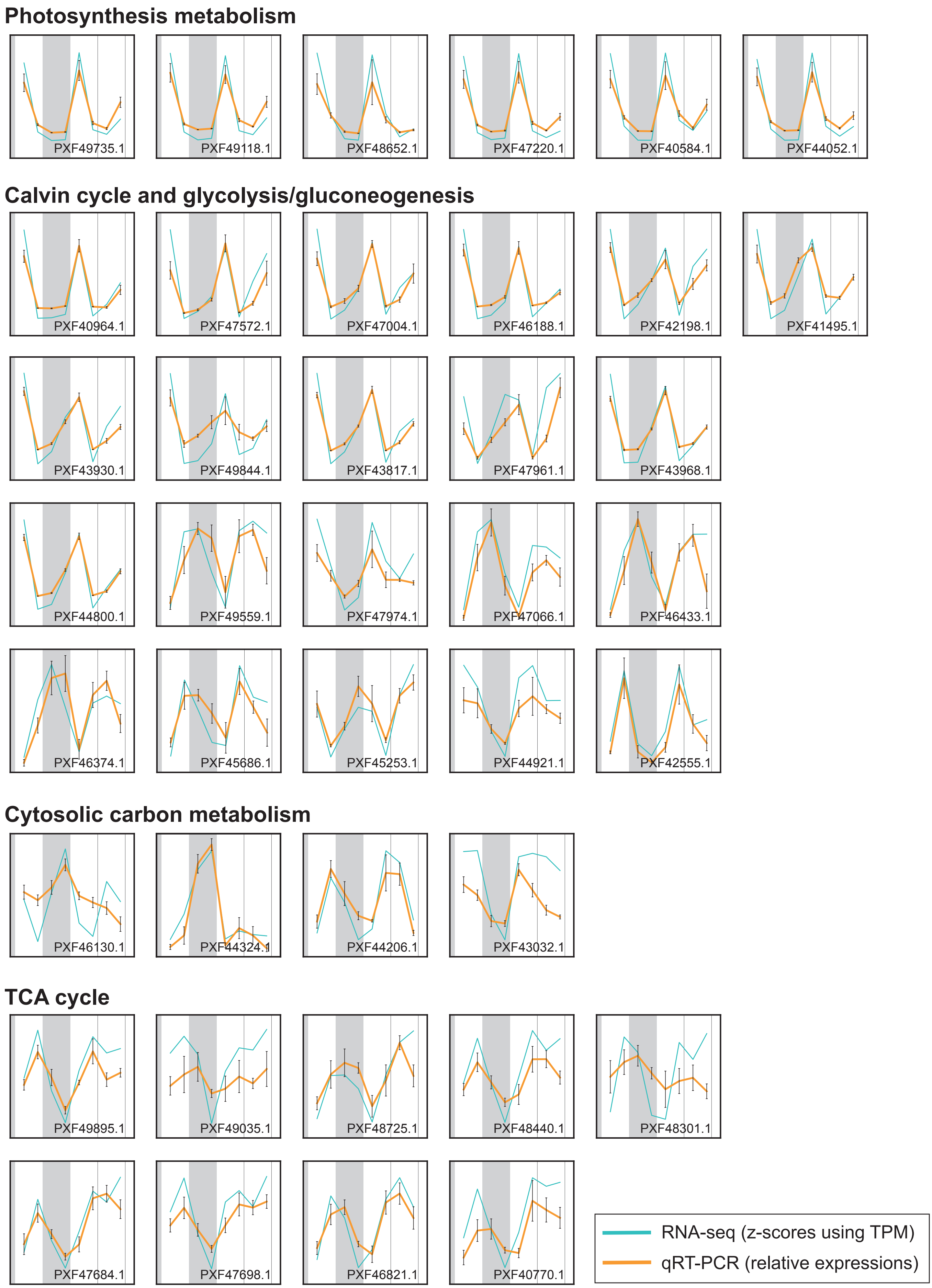

Supplementary Figure S7. Comparison of gene expression patterns using RNA-seq and qRT-PCR. Gene expression patterns of RNA-seq data are shown as z-scores of TPM values (cyan). Gene expression patterns of qRT-PCR results are shown as relative gene expressions (orange) with error bars (standard errors of the means; SEM) using biological triplicates.
